# Genome-wide association studies reveal putative QTLs for physiological traits under contrasting phosphorous conditions in wheat (*Triticum aestivum* L.)

**DOI:** 10.3389/fgene.2022.984720

**Published:** 2022-11-11

**Authors:** Palaparthi Dharmateja, Rajbir Yadav, Manjeet Kumar, Prashanth Babu, Neelu Jain, Pranab Kumar Mandal, Rakesh Pandey, Manoj Shrivastava, Kiran B. Gaikwad, Naresh Kumar Bainsla, Vipin Tomar, S. Sugumar, Nasreen Saifi, Rumesh Ranjan

**Affiliations:** ^1^ Division of Genetics, ICAR-Indian Agricultural Research Institute, New Delhi, India; ^2^ ICAR- National Institute for Plant Biotechnology, New Delhi, India; ^3^ Division of Plant Physiology, ICAR-Indian Agricultural Research Institute, New Delhi, India; ^4^ Division of Environment Science, ICAR-Indian Agricultural Research Institute, New Delhi, India; ^5^ Centre for Quantitative Genetics and Genomics (QGG), Aarhus University, Aarhus, Denmark

**Keywords:** wheat, GWAS, single nucleotide polymorphism, PUE, non-limiting phosphorus and limiting phosphorus

## Abstract

A Genome-wide association (GWAS) study was conducted for phosphorous (P)-use responsive physiological traits in bread wheat at the seedling stage under contrasting P regimes. A panel of 158 diverse advanced breeding lines and released varieties, and a set of 10,800 filtered single nucleotide polymorphism (SNP) markers were used to study marker-trait associations over the eight shoot traits. Principle component analysis separated the two environments (P regimes) because of the differential response of the traits indicating the essentiality of the separate breeding programmes for each environment. Significant variations for genotypic, environmental, and genotype × environment (GEI) effects were observed for all the traits in the combined analysis of variance with moderately high broad sense heritability traits (0.50–0.73). With the different algorithms of association mapping viz., BLINK, FarmCPU, and MLM, 38 unique QTLs under non-limiting P (NLP) and 45 QTLs for limiting P (LP) conditions for various shoot traits were identified. Some of these QTLs were captured by all three algorithms. Interestingly, a Q.*iari.dt.sdw.1* on chromosome 1D was found to explain the significant variations in three important physiological traits under non-limiting phosphorus (NLP) conditions. We identified the putative candidate genes for QTLs namely *Q.iari.dt.chl.1, Q.iari.dt.sdw.16, Q.iari.dt.sdw.9 and Q.iari.dt.tpc.1* which are potentially involved in the mechanism regulating phosphorus use efficiency through improved P absorption due to improved root architectural traits and better mobilization such as sulfotransferase involved in postembryonic root development, WALLS ARE THIN1 (WAT1), a plant-specific protein that facilitates auxin export; lectin receptor-like kinase essentially involved in plant development, stress response during germination and lateral root development and F-box component of the SKP-Cullin-F box E3 ubiquitin ligase complex and strigolactone signal perception. Expression profiling of putative genes located in identified genomic regions against the wheat expression atlas revealed their significance based on the expression of these genes for stress response and growth development processes in wheat. Our results thus provide an important insight into understanding the genetic basis for improving PUE under phosphorus stress conditions and can shape the future breeding programme by developing and integrating molecular markers for these difficult-to-score important traits.

## Introduction

Wheat (*Triticum aestivum* L.), a major cereal crop, meets one-fourth of the protein and one-fifth of the calorie requirement of the total human diet, worldwide. Considering the continuously increasing demand for wheat, its production needs to be increased by at least 50 *percent* by 2050. Additionally, wheat scientists have to look for avenues to increase production under the constraints of declining natural resources and changing climatic conditions (Yadav et al., 2011; Gupta et al., 2016). Globally, the sharp gain in wheat yield was realized with the development and cultivation of lodging tolerant and fertilizer-responsive semi-dwarf wheat varieties during the green revolution era. Since then, yield gain rates are declining in many parts of the world (Yadav et al., 2010). In one of our recent assessments of varieties released over a century-long period (since 1905) for India’s north-western plain zone, the wheat yield has grown at the rate of 0.544% (Yadav et al., 2021).

Globally phosphorus (P), being the essential macronutrient, is among the various major yield-deciding factor for increased crop productivity. P, besides being an essential constituent of genetic material, plays a vital role in photosynthesis, energy transfer, improved root development, starch and sugar transformation, and nutrient flow within the plant system (Pasek, 2008). P-limiting conditions have been reported to be causing stunted growth, reduced effective tillering, thin stems, enhanced root:shoot ratio, and substantial yield reduction in rice and many other cereals (Dobermann and Fairhurst, 2000). At early stages, P deficiency affects the emergence and growth of seedlings (Singh et al., 2013) which in turn leads to decreased root volume, total leaf area, and plant dry weight; however, there is also a significant increase in the density of root hairs and root to shoot ratio (Ahmed et al., 2018). Hence, P feeding during the early stages of wheat growth is crucial for better establishment and any supplementation at later stages would not be compensated in plant growth, which would cause a significant decrease in tiller development and head formation (Grant et al., 2001). Early seedling variations correlate well with the high P uptake. A deeper understanding of the biomass and optimized root traits like root length, root width, root tips number, root diameter, root biomass, and shoot biomass can throw some light on dissecting mechanisms underlying PUE. Hence, exploring the variations for seedlings and their initial biomass accumulation traits under limiting and non-limiting P conditions would provide a better scope for improving elite lines for PUE (YANG et al., 2021). In wheat, the realized yield is also significantly compromised under P deficit conditions worldwide (Zhang et al., 2005). Due to its essential role in physiological activity the high energy requirement of modern high-yielding varieties and the limitation of naturally available P in soil, inorganic P fertilizers are much needed in great quantities in contemporary agriculture. It is anticipated that 52.9 MT of P fertilizers will be used in agriculture by 2030 (Brears, 2015). Nevertheless, large-scale injudicious fertilization raises environmental concerns with unused chemical fertilizers seeping into water systems resulting in eutrophication and affecting marine life adversely (Cassman et al., 2003; Chiou and Lin, 2011). Phosphate rock is a limited and non-renewable global resource with a very short expected exhaustion period (50–200 years) at the current pace of P utilization (Herrera-Estrella and López-Arredondo, 2016), it is high time for its judicious application in agriculture.

Among the various management practices for the rational use of P, the development of P use efficient crop cultivars which come with no additional costs (Heuer et al., 2017) is the most economical. Moreover, for wheat, with the largest area and comparatively lower P utilization rate (10.7%) than rice (13.1%) and maize (11%) (Ma et al., 2011), focuses on the fast-track approaches to identify P efficient cultivars is very essential (Dharmateja et al., 2021). Productivity gains with low P-application by growing P-efficient cultivars would pave the way to meet future global food requirements in an eco-friendly, economically feasible, and socially sustainable manner. Moreover, compared to modern wheat varieties which are bred under optimal P conditions, landraces and traditional local germplasm have greater PUE under unfertilized and P-fixed soil due to their natural selection in the evolutionary process and adaptation to P-deficient conditions (Wissuwa and Ae, 2001). The use of such genetic variability would help to improve modern cultivars for these traits.

To develop P-efficient wheat varieties, knowledge about fundamental traits and their associated genomic regions controlling P uptake and utilisation is of utmost importance. The advances in genomics in wheat over the last decade enabled breeders to identify the genetic markers liked to traits of interest and to facilitate integrating them into the breeding lines for various difficult-to-measure target traits. High throughput genotyping arrays have revolutionized marker-trait-associations studies and have helped to fine-map the target genomic regions. PUE-related traits are largely governed by several minor genes with small cumulative effects. Previously, QTLs for P-deficiency-tolerance have been identified through linkage mapping in biparental wheat populations (Yuan et al., 2017). But, considering large minor genes affecting PUE and its complexity association mapping by exploring more than two possible alleles at various genomic regions in a genetic panel consisting of lines with a diverse pedigree will have more probability of capturing factors explaining the phenotypic variation for P use efficiency than the bi-parental mapping population. Accordingly, Genome-wide association studies (GWAS) have effectively explored allelic variations for P-deficiency tolerance in *Aegilops tauschii* (Liu et al., 2015), Arabidopsis (Bouain, Doumas, and Rouached, 2016) and soybean (Ning et al., 2016).

When measuring a trait response of P among genotypes, precise phenotyping becomes the major limiting factor. Many researchers have struggled to precisely quantify the impact of P on plant growth modifications. Testing genotypes against a particular concentration of measured P is not a feasible task under field conditions. This approach has been limited due to the uncontrollable natural contribution of P through inorganic supply from manures and microbial interaction, and moreover, it is difficult to maintain a uniform supply of P throughout the growing period in the soil. To overcome this limitation and to measure genotypic responses to sole P concentration variations, hydroponic systems are being used in many crops to accurately quantify the P and its uptake by plants. Due to their subterranean nature, these systems have been considered to be the best way to study the root system modifications which would also permit for precise estimation of root and shoot parameters. The simplified hydroponics systems based on aerated nutrient solutions with various levels of P with an option for complete replacement of nutrient solutions at fixed intervals were reported (Byrne et al., 2011; Gong et al., 2011) and successfully used in PUE studies. Hydroponic systems can also prove to be an efficient approach for screening large populations under different nutrient conditions and less amount of space.

In recent decades, several quantitative trait loci (QTLs) for PUE and related traits have been identified and mapped on all 21 chromosomes in wheat under hydroponic culture trials (Guo et al., 2012; Zhang and Wang, 2015). In addition, the phosphorus uptake (PupE), the utilization efficiency (PutE), and the response of morphological traits under different P levels were investigated in hydroponic culture (Yuan et al., 2017b). Categorization of wheat germplasm for PUE was done based on P use efficiency parameters, recorded on genotypes grown in hydroponics with two P regimes (Bilal et al., 2018). PUE-enhanced crop cultivars can have more growth and biomass for the same quantity of P taken up at a given time (Rengel and Marschner, 2005). However, having low heritability for this trait, breeding for PUE is complex and influenced by multiple physiological processes, besides enormous environmental impacts (Heuer et al., 2017). Therefore, dissecting its inheritance patterns into underlying genetic factors at the genomic level can circumvent these environmental influences. With this background, in our current study, we performed a GWAS on a set of high-yielding genotypes with diverse pedigree/parentage to identify marker-trait associations (MTAs) for P use efficiency with a 35 K breeders Affymetrix SNPs array for genotyping. More interestingly, this study has been planned on a panel of indigenously bred, high-yielding advanced breeding lines so that the subsequent gain in PUE can be built upon among these elite lines without compromising yield gain. In addition, the insight gained in understanding P use efficiency among selected genotypes and identified QTNs (Quantitative traits nucleotides) governing PUE will pave the way to develop better genotypes for this challenging-to-score and complex-to-study trait in wheat.

## Materials and methods

### Plant materials and hydroponic culture

A set of 158 advanced bread wheat lines of spring type with great diversity in their pedigree and lineage were grown under non-limited and limited phosphorus conditions in hydroponics. This set also included released varieties with their wider adaptation to different agro-climatic conditions (Supplementary Table S1). The phenotypic assay under hydroponics was conducted at National Phytotron Facility at the ICAR-Indian Agricultural Research Institute, New Delhi, India. Individual genotypes were grown under a controlled environment with typical growing parameters of 12°C–22°C day/night temperature, 10 h day length of photoperiod, and 70% relative humidity were maintained throughout the growing period. The seeds of individual genotypes were germinated in a separate petri dish with blotting paper containing sufficient moisture for 5–6 days. The uniform-sized five-day-old seedlings were transferred to the hydroponics tank system consisting of a tray of 18-litre capacity covered with a black ceramic lid. The basal nutrient solution used in the hydroponics experiment consisted of (NH_4_)_2_SO_4_·H_2_O (1 mmol/L), Ca(NO_3_)_2_·4H_2_O (1 mmol/L), KCl (1.8 mmol/L), MgSO_4_·7H_2_O (0.5 mmol/L), CaCl_2_ (1.5 mmol/L), H_3_BO_3_ (1 μmol/L), CuSO_4_·5H_2_O (0.5 μmol/L), ZnSO_4_·7H_2_O (1 μmol/L), MnSO_4_·H_2_O (1 μmol/L), FeEDTA (100 μmol/L), and (NH_4_)_6_Mo_7_O_24_·4H_2_O (0.1 μmol/L), and two levels of P were maintained using KH_2_PO_4_ as Non limiting P (0.2 mmol/L) and limiting P (0.02 mmol/L) (Yuan et al., 2017; Bilal et al., 2018). The nutrient solution was continuously aerated, and the pH was maintained between 6 and 6.5 using 1 M KOH and 1 M HCL. The consistent nutrient provision is supported by basal solution supplanted with fresh solution every 4 days.

### Advanced phenotyping for phosphorous-responsive traits

The experimental material was tested in the completely randomized design (CRD) design with three replications, and five plants of thirty-day-old seedlings (Zadok’s scale: growth stage 29) (Zadoks, Chang, and Konzak, 1974) from each replication under NLP and LP hydroponic conditions were taken out for recording the observations on traits under study. The root and shoots of the individual plant were separated carefully using scissors. LI-COR 3000 (Lincoln, NE, United States) leaf area meter was used to measure the total leaf area (TLA, m^2^/plant). The Chl content (µmmol/m^2^) was measured with SPAD (Minolta Camera Co., Osaka, Japan) (Minolta 1989). The SPAD readings are measured based on the transmission of red light (650 nm) and infrared light (940 nm), in which red light is absorbed by chlorophyll (Xiong et al., 2015). The Chl data with SPAD was recorded on a fully-opened, well-developed topmost leaf of 30 days old seedlings in each replication on five plants with an average of three readings between 11 a.m. and 12:30 p.m. The variability in SPAD reading is influenced by environmental factors, diurnal variation, and genotypic differences in Chl content under treatment. Therefore, we tried to keep the SPAD reading variability to the minimum level by taking the observations under controlled conditions and with a minimum time difference in data recording. The separated plant parts, i.e., shoot and roots, were dried in a hot air oven at 60°C until stable dry biomass was obtained, then measured for shoot dry weight (SDW, g/plant) and root dry weight (RDW, g/plant). The RSR was calculated as shoot-dry weight (SDW) to root-dry weight (RDW) ratio. The combined shoot and root-dry weight of samples were considered as total dry weight (TDW, g/plant). To estimate the P content, the fine-grind and dried sample of each plant was digested in a diacid mixture (HNO_3_:HCLO_4_) until a clear solution was obtained. The total P (mg) was analyzed using the vanadium molybdate yellow colourimetric method (Ma et al., 2021a). The tissue phosphorus content multiplied by total dry weight is used to calculate the total P uptake by the plant (TPU, mg) (Wang et al., 2017). The P utilization efficiency (PUtE, dry weight (g)/P (mg)) was calculated using the following formula under both NLP and LP conditions (Wang et al., 2017).

PUtE [dry weight (g)/P (mg)] = Total dry weight/total P uptake by plant.

### Phenotypic data analysis

The presence of outliers in the data was confirmed by boxplot analysis and with the Z score test. The phenotypic data generated through trials in a completely randomised design were subjected to the analysis of variance (ANOVA) with STAR version 2.1.0 (Statistical Tool for Agricultural Research) an R-based software (Gulles et al., 2014). The variability analysis and adjusted means were calculated as best linear unbiased predictions (BLUPs) considering the replication and genotypes as random effects separately for each P regimes using META-R version 6.0 (Alvarado et al., 2020). The adjusted means of replicates in the trial were obtained by fitting mixed linear models (MLM) using the equation.

$$Y_{ik}=\mu +R_{i}+G_{k}+\epsilon_{ik}$$

where $Y_{ik}$ is the trait of interest; μ is the mean effect; $R_{i}$ is the effect of the *i*th replicate; $G_{k}$ is the effect of the *k*th genotype; $\epsilon_{ik}$ is the error associated with the *i*th replication and the *k*th genotype, which is assumed to be normally and independently distributed, with mean zero and homoscedastic variance σ^2^. The genotypes and replicates were considered random effects to calculate adjusted means, while P regimes as a fixed effect. The broad-sense heritability was estimated using the formula.

$$H^{2}=\frac{\sigma_{g}^{2}}{\sigma_{g}^{2}+\sigma_{e}^{2}/n\;reps}$$

Where $\sigma_{g}^{2}$ is the genotypic variance; $\sigma_{e}^{2}$ is the error variance and n reps is the number of replications. The broad-sense heritability estimated the quality of the breeding program for the traits and the environments. The LSD with type I error, *α* = 0.05 of the level of significance, was calculated using the formula:

$$LSD=t_{\left(1-0.05,dferror\right)}\times ASED$$

Where *t* is the cumulative “Student’s *t*-test” distribution; *df error* is the degrees of freedom for the variance of error, and ASED is the average standard error of the differences between pairs of means. And the coefficient of variation (%) is calculated using the formula:

$$CV\left(\%\right)=100\times \frac{ASED}{grand\;mean}$$

The phosphorus deficiency tolerance coefficient (PDTC) was calculated as the ratio of LP over NLP treatment (Li et al., 2015). The correlations were calculated as simple pairwise Pearson’s correlations among traits. Further, principal component analysis (PCA) was performed to identify the number of principal components required to explain the variation across the environments and to study the relationship between traits and the effect of treatments using R package version 4.0.1 (R core Team, 2022).

### Genome-wide studies to establish marker traits associations

Genomic DNA was extracted by following the CTAB method (Doyle and Doyle, 1987) and the quantity and quality of DNA were estimated by using an agarose gel electrophoresis approach and UV spectrophotometer. The 35 K Axiom^®^ Wheat Breeder’s Array (Affymetrix UK Ltd., United Kingdom) was used for genome-wide association analysis**.** Monomorphic markers, markers with >30% missing data, 5% minor allele frequency, and greater than 20% heterozygosity were removed. A filtered set of 10,800 highly informative SNP markers was used for GWAS. Population structure among the 158 genotypes was determined by using STRUCTURE v. 2.3.4 (Pritchard, Stephens, and Donnelly, 2000). Population structure was generated the over 10,000 length of burnin period and 100,000 MCMC reps with three iterations. The optimum population number (K) value was determined by the *ad-hoc*, delta K method (Evanno et al., 2005). Constellation plot plotted by using the Ward method in JMP v.14 (Lehman et al., 2005). Linkage disequilibrium (LD) analysis was performed on a sub-population basis, using the LD function in TASSEL (Bradbury et al., 2007) and draw the LD plot with R software version 4.2.1 (R core Team 2022). Associations between genotypic and phenotypic data were evaluated in GAPIT version 3.0 (Wang et al., 2021) using MLM, BLINK and FarmCPU algorithms. The Mixed linear model (MLM) is carried out by taking into account both the Q-matrix and the K-matrix (the kinship matrix expressing family relatedness among the genotypes) (Yu and Buckler, 2006; Tadesse et al., 2015). The FarmCPU makes use of stepwise regression [fixed-effect model (FEM)] and the mixed linear model (MLM) (X. Liu et al., 2016). The FEM is used to evaluate genetic markers, while the REM is used to control false positives by including associations or pseudo-quantitative trait nucleotides as covariates in the model. The Bayesian-information and Linkage-disequilibrium Iteratively Nested Keyway (BLINK) is very computationally efficient and reliable, and algorithum is based on the fixed effect model involving the Bayesian information criteria and linkage disequilibrium information (Huang et al., 2019).

The kinship matrix was calculated from the 10,800 markers, and QQ and Manhattan plots were generated to evaluate the results. The adjusted *p*-value threshold of significance was corrected for multiple comparisons according to the false-discovery rate (FDR) with cut-off ≤ 0.05 with Benjamini and Hochberg method (https://tools.carbocation.com/FDR) (Benjamini and Hochberg, 1995). After the identification of MTAs, an *in silico* search of the putative candidate genes with their annotated functions was conducted in the Ensembl Plants database (http://plants.ensembl.org/index.html) of the bread wheat genome (*Triticum aestivum* L.). The Chinese spring wheat cultivar was utilized in making of genome assembly IWGSC-refseq version 1.0. The data from CerealDB was utilized to locate the genes in the wheat version hosted on Ensembl. The gene expression atlas was used to analyse the expression of detected putative candidate genes (http://www.wheat-expression.com/) (Borrill et al., 2016).

## Results

### Phenotypic variation and heritability for traits under non-limiting phosphorus and limiting phosphorus conditions

**FIGURE 1:**
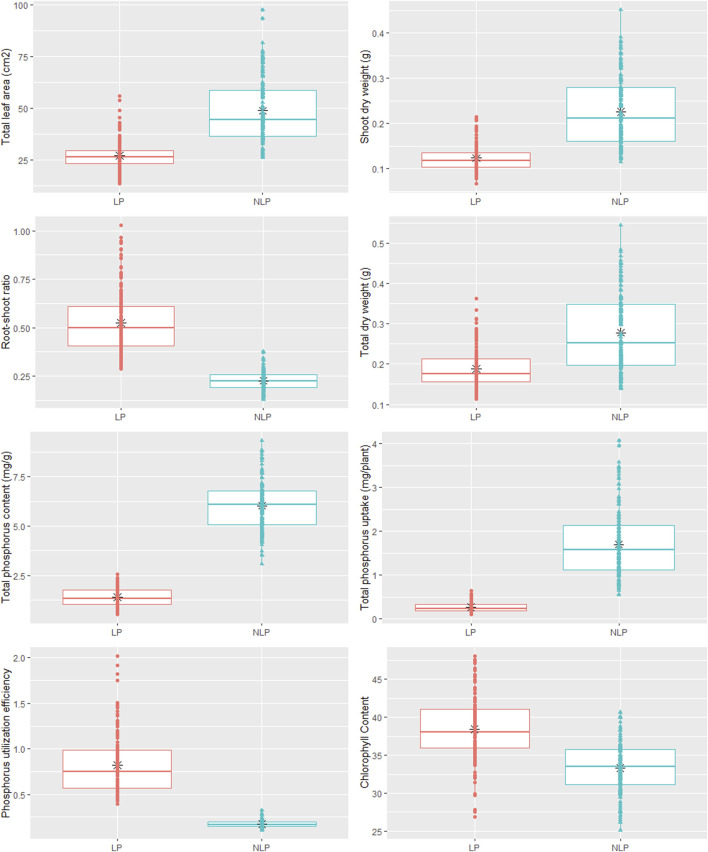
Box plot showing phenotypic variation in wheat genotypes in non-limiting and limiting phosphorus conditions.

Phenotypic data on P-responsive traits among the 158 wheat genotypes were recorded under NLP and LP conditions. The combined analysis of variance indicated significant variations due to the genotype (G), different P-treatment (T) regimes (NLP and LP), and genotype-P treatment interaction effect (G × T) for all the traits. The broad-sense heritability expressed as the proportion of total phenotypic variance for each trait under this study was moderate, ranging from 0.50 (PUtE) to 0.73 (TDW), indicating the involvement of both genetic and environmental variations in governing these traits related to P-response (Table 1). The boxplots of various traits under study have been presented in (Figure 1), with mean values as “*”. The traits namely TLA, SDW, TDW, TPU, and TPC were higher in NLP than LP, while RSR, Chl content, and PUtE were higher in LP. The spread of variability for all traits under study was higher in NLP except for PUtE, which was limited. The overall coefficient of variation was ranging from 3.10% to 13.70% across the traits tested. Limiting the phosphorous supply reduced TLA, SDW, TDW, TPC, and TPU. TLA ranged from 18.94 to 97.55 m^2^/plant in NLP and 13.32–55.65 m^2^/plant in LP while SDW ranged from 0.114 to 0.451 g/plant for NLP and 0.067–0.214 g/plant in LP. TDW ranged from 0.139 to 0.545 g/plant under NLP condition against 0.111–0.362 g/plant in LP. A higher value was observed for TPC and TPU in a P-rich environment with a range of 3.07–9.33 against 0.499–2.54 for LP. TPU varied from 0.541 to 4.075 under NLP and 0.092–0.647 in LP. In contrast, under LP, increased values were observed for Chl, RSR, and PUtE. Chl varied from 25.14 to 40.66 μm mol/m^2^ for NLP and 23.42–48.06 μm mol/m^2^ for LP, with a mean value higher in LP than NLP (Table 2). Similarly, a higher ratio was observed for RSR in LP across all genotypes tested, with a range of 0.287–1.03 in LP. The PUtE was found higher under limiting phosphorus with a range of 0.396–2.02 against 0.107–0.326 under NLP. The phosphorus deficiency tolerance coefficient (PDTC) was calculated as the ratio of different trait mean values in LP over NLP for a better and quicker understanding of trait response under two conditions. The traits like Chl (1.15), RSR (2.33), and PUtE (4.73) with >1 PDTC value indicated their better expression under LP. The remaining traits TLA (0.556), SDW (0.550), TDW (0.677), TPC (0.231), TPU (0.154) expressed better with availability of sufficient phosphorus as their PDTC value was < 1.

**TABLE 1:** Analysis of variance and heritability for the traits under non-limiting and limiting phosphorus.

	Source of variation	TLA	Chl	SDW	RSR	TDW	TPC	TPU	PUtE
MSS	Phosphorus (P)	112,084.50**	6138.24**	2.47**	21.04**	1.93**	5089.56**	490.76**	99.68**
	Genotype (G)	515.10**	57.78**	0.0132**	0.0520**	0.026**	2.892**	0.9996**	0.1623**
	G*P	414.11**	28.44**	0.0074**	0.0328**	0.0093**	2.60**	0.8431**	0.1628**
	Error	26.89	1.42	0	0.0005	0.0001	0.0096	0.0019	0.0014
V_G_		122.05	14.09	0.0033	0.0129	0.0064	0.7206	0.2494	4.02E-02
V_G_ P_H_		193.61	13.51	0.0037	0.0162	0.0046	1.29	0.4206	8.07E-02
V_E_		26.89	1.42	0	0.0005	0.0001	0.0096	0.0019	1.40E-03
V_P_		225.58	21.20	0.0052	0.0211	8.8E-03	1.37	0.4602	0.0809
Grand mean		37.85	35.86	0.1751	0.3749	0.233	3.71	0.9807	0.4982
CV (%)		13.70	3.32	3.53	5.92	3.1	2.64	4.43	7.6
LSD		7.27	3.07	0.0458	0.0887	0.0622	0.4138	0.2929	4.23E-06
Heritability		0.54	0.66	0.64	0.61	0.73	0.53	0.54	0.50

TLA, total leaf area; Chl, chlorophyll content; SDW, shoot dry weight; RSR, root:shoot ratio; TDW, total dry weight; TPC, total phosphorus content; TPU, total phosphorus uptake; PUtE, phosphorus utilization efficiency; V_G,_ genotypic variance; V_E_, error variance; V_P,_ total phenotypic variance; CV, coefficient of variation; LSD, Least Significant Difference.

*p < 0.05; **p < 0.01 and ***p < 0.001 include significance in tables.

**TABLE 2:** Measures of variability and phosphorus deficiency tolerance coefficient (PDTC) for the traits under study.

Trait	NLP		LP		Mean		PDTC
	MIN	MAX	MIN	MAX	NLP	LP	
TLA	18.94	97.55	13.32	55.65	48.48	26.95	0.556
Chl	25.14	40.66	23.42	48.06	33.31	38.37	1.15
SDW	0.114	0.451	0.067	0.214	0.226	0.124	0.550
RSR	0.122	0.377	0.287	1.03	0.225	0.523	2.33
TDW	0.139	0.545	0.111	0.362	0.278	0.188	0.677
TPC	3.07	9.33	0.499	2.54	6.02	1.39	0.231
TPU	0.541	4.075	0.092	0.647	1.70	0.261	0.154
PUtE	0.107	0.326	0.396	2.02	0.174	0.822	4.73

TLA, total leaf area; Chl, chlorophyll content; SDW, shoot dry weight; RSR, root:shoot ratio; TDW, total dry weight; TPC, total phosphorus content; TPU, total phosphorus uptake; PUtE, phosphorus utilization efficiency; MIN, minimum value; MAX, maximum value; PDTC, phosphorus deficiency tolerance coefficient.

### Trait correlations and principal component analysis

**FIGURE 2:**
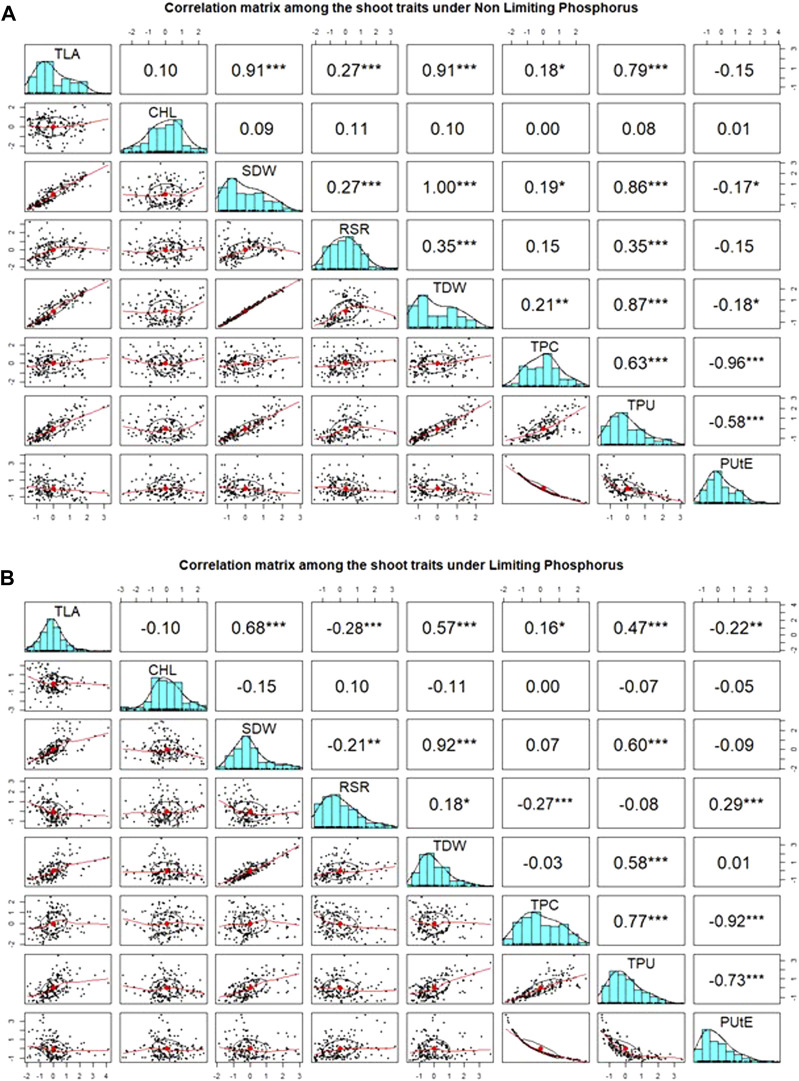
Association between measured traits and distribution among wheat genotypes under non-limiting (NLP) **(A)** and limiting (LP) phosphorus **(B)** conditions. TLA, total leaf area; Chl, chlorophyll content; SDW, shoot dry weight; RSR, root:shoot ratio; TDW, total dry weight; TPC, total phosphorus content; TPU, total phosphorus uptake; PUtE, phosphorus utilization efficiency.

**FIGURE 3:**
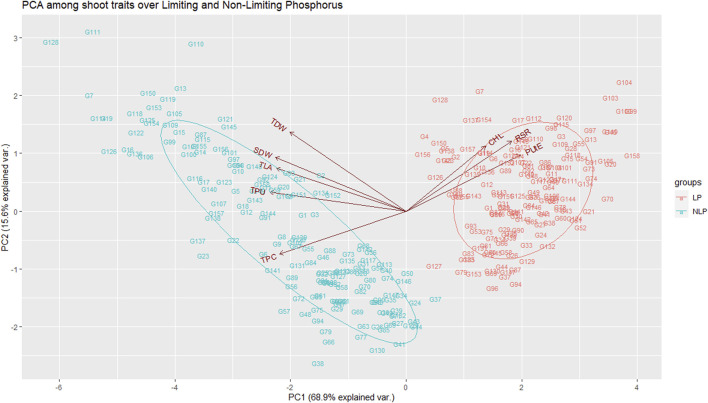
Principal component analysis among the traits over non-limiting and limiting conditions. TLA, total leaf area; Chl, chlorophyll content; SDW, shoot dry weight; RSR, root:shoot ratio; TDW, total dry weight; TPC, total phosphorus content; TPU, total phosphorus uptake; PUtE, phosphorus utilization efficiency.

Pearson’s pairwise correlation coefficient was calculated among various traits under both environments. Chl was not associated with any other traits, neither in NLP nor in LP. The most significant (*p < 0.001*) correlations were observed between TLA and SDW under both conditions (Figures 2A,B). Interestingly, RSR was positively correlated with TLA under NLP and negatively under LP conditions. Very strong positive correlations were observed for TLA with SDW, TDW and TPU; SDW with TDW and TPU and TPU with TDW and TPC under both NLP and LP conditions indicating their strong influence on each other. The TDW showed a significant positive correlation with TPC in the NLP condition, and a non-significant correlation was observed in the LP condition. TPC had a significant positive correlation with TPU, and it was interesting to note that both of these showed a strong negative association with PUtE under both environments. In addition, the PUtE was also negatively associated with most of the traits in NLP and LP conditions except for a significant positive association with RSR in LP conditions. PCA-based grouping of traits over LP and NLP conditions indicated that the first two principal components (PC1 and PC2) had explained 84.5% (68.9% and 15.6%) of the total variation (Figure 3). The existence of a high G x T effect indicates that these treatments significantly affected the traits studied. PCA analysis showed that TLA was highly dependent on SDW, TDW, TPC, and TPU and least dependent on Chl, RSR, and PUtE. Similarly, Chl was dependent on RSR and, PUtE, with the least dependence on SDW, TDW, TPC, and TLA. The results clearly depicted in the figure that the trait expression varies with the availability of P. The genotypes under NLP condition exhibit better expression for TLA, SDW, TDW, TPC, and TPU, and in the LP condition exhibit higher expression for Chl, RSR, and PUtE. Along with the treatment, genotypes are separated and all eight traits fall into two distinct groups. The correlation between these traits is represented by the angle between their vectors. The traits TLA, SDW, TDW, TPC, TPU and Chl, RSR, and PUtE are highly correlated because the angle between them is very small (acute angle i.e., < 90°). But there is a significant crossover between the traits of these distinct groups because the presence of a wider angle (i.e., > 90°).

### Genome-wide association mapping of phosphorous-responsive physiological traits

**FIGURE 4:**
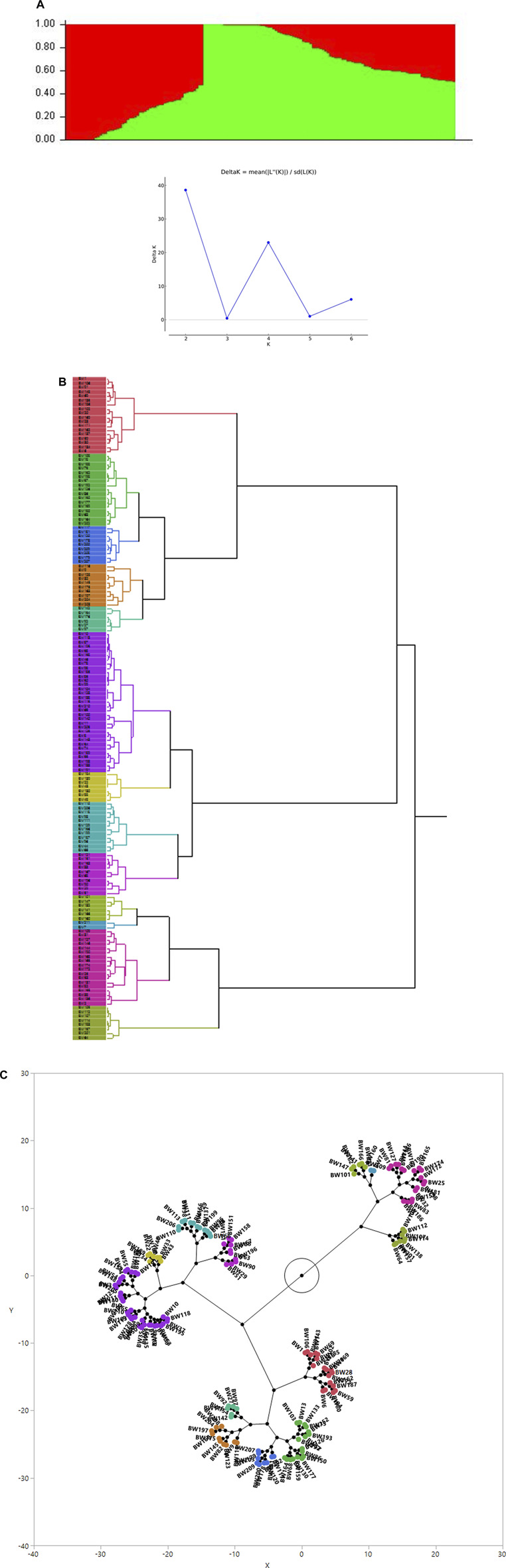
**(A)** Population genetic structure plot in association panel of 158 wheat genotypes (optimal population number K = 2 with two different colours) and Delta K plot depicting peak at K = 2. **(B)** Dendrogram **(C)** Constellation plot (showing three groups) using the Ward method in JMP v.14.Figure.

**FIGURE 5:**
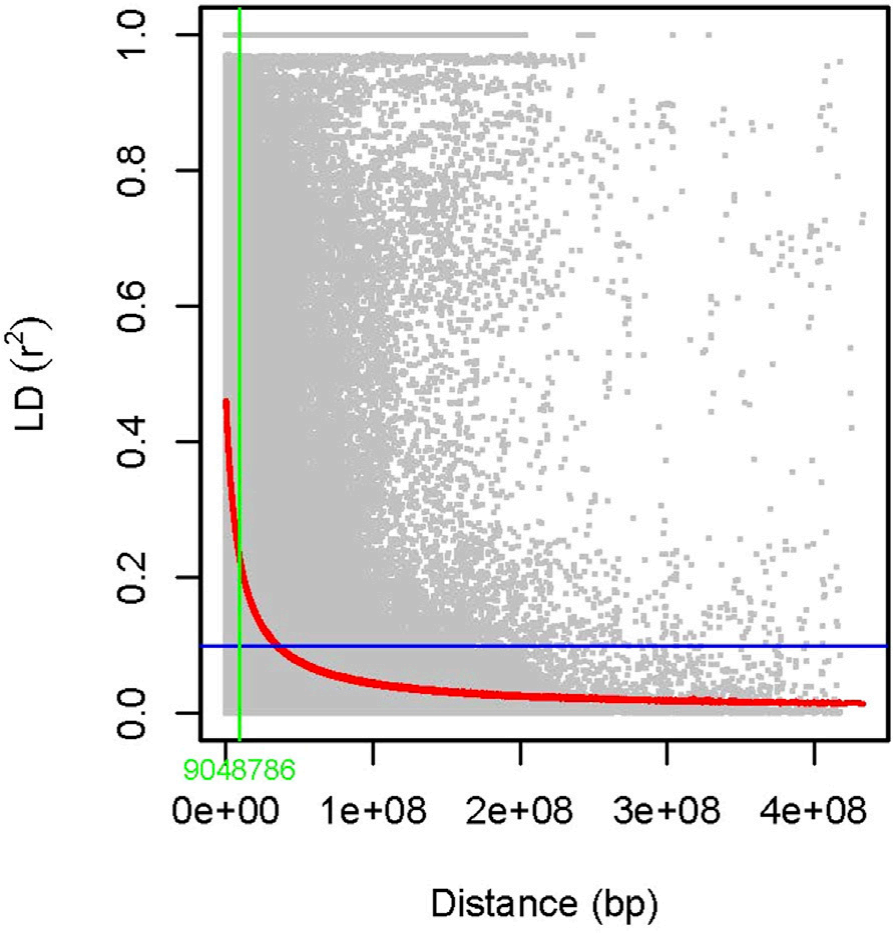
Scatter plot showing linkage disequilibrium (LD) decay estimated by plotting (*r* ^2^) against genetic distance (bp).

**FIGURE 6:**
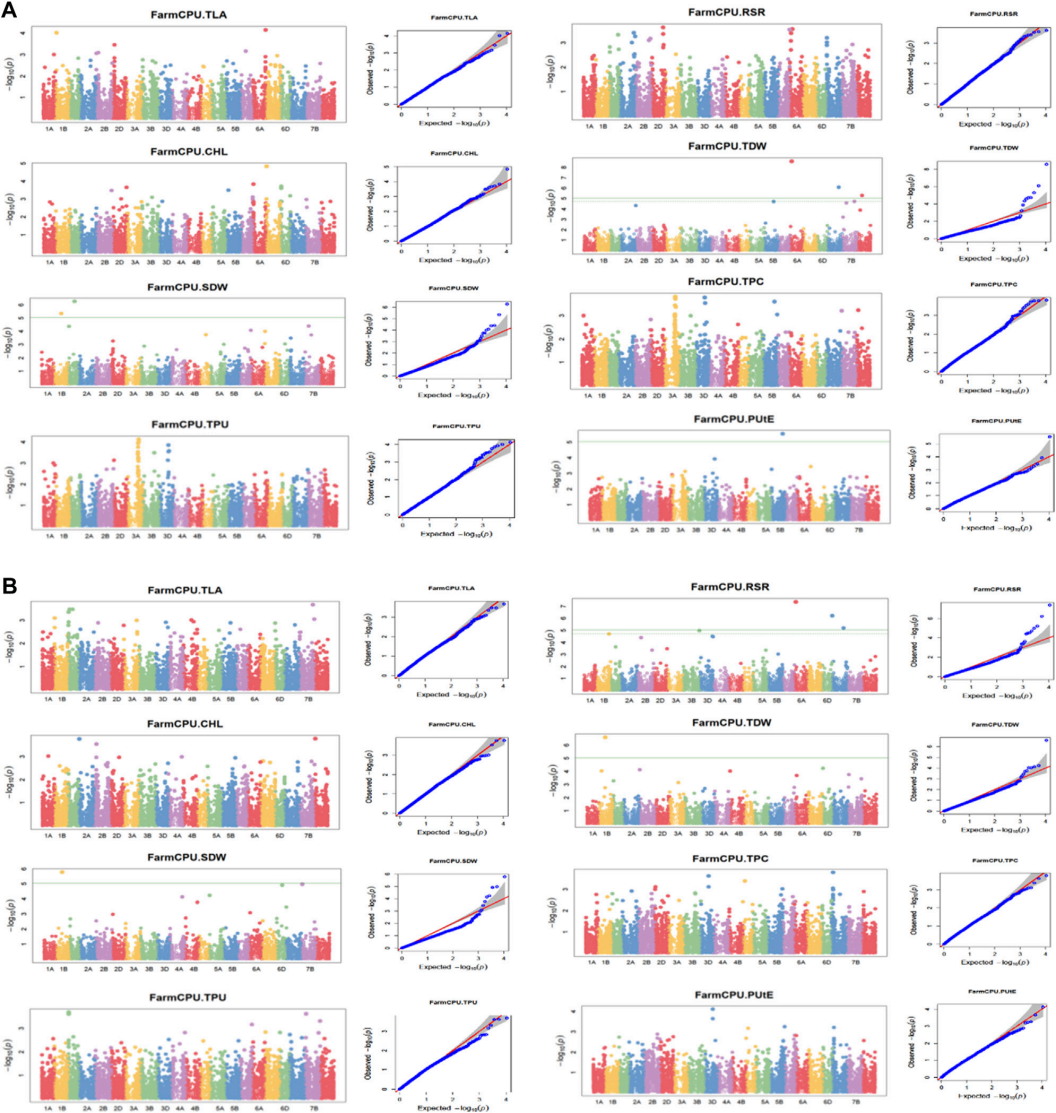
**(A)** (NLP) and **(B)** (LP): Manhattan plots and Q-Q plots (using FARM CPU) for Phosphorus use efficiency traits. TLA, total leaf area; Chl, chlorophyll content; SDW, shoot dry weight; RSR, root: shoot ratio; TDW, total dry weight; TPC, total phosphorus content; TPU, total phosphorus uptake; PUtE, phosphorus utilization efficiency.

The association analysis was performed using three algorithms (Blink, FarmCPU, and MLM) for the physiological traits studied under NLP and LP conditions. A total of 10,800 SNP’s derived from the SNP chip array for 158 genotypes were used for mapping and their distribution across the chromosomes was depicted (Supplementary Table S2). Among association mapping panel population structure was calculated by using STRUCTUREv2.3.4 software (Figure 4A). The population structure showed a sharp peak at K = 2 when the clusters were plotted against ΔΚ indicating two subgroups in the population and the dendrogram, constellation plot revealed two major groups, thus results were further confirmed (Figures 4B,C). A set of 10,800 high-quality SNP markers were distributed across the genome with the B genome (4041) having the highest number of markers, followed by the A genome (3409) and the D genome (3350) respectively. According to chromosome-wise distribution, chromosome 2B (749) had the most markers mapped, followed by chromosome 2D (707). The least number of markers were found on chromosomes 4D (180) and 6B (277). The LD was estimated by calculating the squared correlation coefficient (*r2*) for all the 10,800 markers. The genome-wide LD decay with physical distance, the LD decay to its half at 9.04 Mb for the whole genome (Figure 5). The −log *p*-value = 3.5 is considered as threshold to call the MTAs as significant associations and corrected according to the false-discovery rate (FDR cut-off ≤ 0.05). A total of 83 QTLs were detected, among them 45 in NLP and 38 in LP conditions identified for the eight traits under study. With different algorithms under NLP conditions, 19 (BLINK), 32 (Farm CPU), 9 (MLM) QTLs were identified, and similarly, under LP conditions, 26 (BLINK), 34 (FARM CPU), and 21 (MLM) QTLs were detected (Supplementary Table S3) (Figures 6A,B; Supplementary Figures S1A–D). Fourteen QTLs (4 in NLP and 10 in LP) were detected in all the algorithms (Supplementary Table S4). Four QTLs were detected for TLA, located one each on chromosome 7B, 1D, 6A, and 1B across both the conditions. In NLP condition *Q.iari.dt.tla.1* and *Q.iari.dt.tla.2* were detected on chromosome 7B and 1D with their associated SNPs AX94470386 and AX94765690 with −log10 *p*-value with 3.70 and 3.5 respectively. Two QTLs namely *Q*.*iari.dt.tla*.*3* and *Q.iari.dt.tla*.*4* with their associated SNPs as AX94770913 and AX95190390 with −log10 *p*-value ranging from 4.2 to 4.6 and 4.0 to 4.3, respectively, were detected in LP conditions on chromosome 6A and 1B. For Chl content, three QTLs, namely *Q.iari.dt.chl.1*, *Q.iari.dt.chl.2*, and *Q*.*iari.dt.chl.3* associated with SNPs AX94832883, AX94676652, and AX95105278 were detected in NLP conditions on chromosomes 7D, 2A, and 2B. Under LP condition, *Q.iari.dt.chl.4, Q.iari.dt.chl.5, Q.iari.dt.chl.6, Q.iari.dt.chl.7,* and *Q.iari.dt.chl.8* were detected on chromosomes 6B, 6A, 6D, 2D, and 6D for Chl content with their associated SNPs AX94597699, AX95241386, AX94702861, AX94622481, and AX95230097 with −log10 *p*-value ranging from 3.5 to 4.8. Seven QTLs under NLP and thirteen QTLs under LP were detected for SDW with a range of −log10 *p*-value from 3.74 to 8.13. For RSR, thirteen QTLs in NLP and seven QTLs in LP conditions were detected. SDW is very strongly associated with TDW, seven QTLs in the NLP condition and twelve QTLs in the LP condition were detected to explain the variation for TDW. For TPC five QTLs, two each on chromosomes, 3A and 3 D and one on 7A were identified under both the treatments. Under NLP conditions, three QTLs and in LP four QTLs were detected for the trait TPU. However, only two QTLs were found to explain the variation for PUtE in both NLP and LP conditions.

Interestingly, a QTL *Q.iari.dt.sdw.1* associated with SNP AX94514240 located on chromosome 1D was found to explain the significant variation for all three traits, namely SDW, TDW, and TPU under NLP conditions (Table 3). Though SDW is part of TDW, only four common QTLs viz. *Q*.*iari.dt.sdw.2* associated with SNP AX94646448 on 1B; *Q.iari.dt.sdw.8* and *Q*.*iari.dt.sdw.14* on 7B associated with SNPs AX94638774 and AX94626370, under NLP condition, and a QTL *Q*.*iari.dt.sdw.20* associated with SNP AX94456805 detected on chromosome 2D under LP condition explained the variation for both traits. The traits like SDW and TPU shared a common QTL named *Q*.*iari.dt.sdw.3* present on chromosome 7B and associated with SNP AX94396598 was detected to explain the variation for traits SDW and TPU, simultaneously under NLP condition only. Another QTL named *Q*.*iari.dt.rsr.1* on chromosome 6A and associated with SNP AX94475513 was associated with two traits, RSR and TDW under both conditions. In the NLP condition, TPC shared common QTLs named *Q*.*iari.dt.tpc.2* and *Q*.*iari.dt.tpc.3* which were associated with SNPs AX94584110 and AX94397869 on the chromosome 3A and 3D with the other traits PUtE and TPU, respectively.

**TABLE 3:** List common QTLs detected across the traits and treatments.

S. No.	Traits	Treatment	QTLs	Chr	Traes ID	Function	References
1	SDW, TDW, TPU	NLP	*Q.iari.dt.sdw.1*	1D	TraesCS1D02G029900	Leucine-rich repeat domain superfamily, NB ARC, P-loop containing nucleoside tri phosphate hydrolase	Flor (1956), Hammond-Kosack and Jones (1997)
2	SDW, TDW	NLP	*Q.iari.dt.sdw.2*	1B	TraesCS1B02G167700	Protien kinase like-domain super family	Sondergaard et al. (2004)
3	SDW, TPU	NLP	*Q.iari.dt.sdw.3*	7B	—	—	—
4	SDW, TDW	LP	*Q.iari.dt.sdw.8*	7B	—	—	—
5	SDW, TDW	LP	*Q.iari.dt.sdw.14*	7B	TraesCS7B02G149200	Heat shock protein 90, cellular response to heat, protein stabilization	Kumar et al. (2020); G. Wang et al. (2011)
6	SDW, TDW	LP	*Q.iari.dt.sdw.20*	2D	TraesCS2D02G584900	Tubby-like F-box protein	Hong et al. (2016)
7	RSR, TDW	NLP and LP	*Q.iari.dt.rsr.1*	6A	TraesCS6A02G095100	F-box-like domain super family, FBD domain, Leucine-rich repeat domain superfamily, Leucine-rich repeat 2	Lechner et al. (2006)
8	TPC, PUtE	NLP	*Q.iari.dt.tpc.2*	3D	TraesCS3D02G267000	Glycerolipid biosynthetic process, diacylglycerol O-acyltransferase activity	Hernández, M. L., 2012
9	TPC, TPU	NLP	*Q.iari.dt.tpc.3*	3A	—	—	—

### The annotation of putative candidate genes functions

Based on the physical locations of associated SNPs and their linked QTLs, an attempt was made to identify the candidate genes harbouring the associated SNPs (Table 4). The SNP AX94470386 linked to QTL *Q*.*iari.dt.tla.1* of TLA was found in the gene TraesCS7B02G382300 coding for ATPase-associated with cation transmembrane transporter activity. Similarly, the SNP AX94765690 is associated with QTL *Q.iari.dt.tla.2* of TLA was found within the gene TraesCS1D02G075500 coding for basic region/leucine zipper protein; a positive regulator of transcription. The SNP AX94770913 linked to QTL *Q.iari.dt.tla.3* of TLA was also found in TraesCS6A02G406500 gene transcribing ribosomal protein L9 in bacteria/chloroplast. The SNP AX94832883 is linked to QTL Q.iari.dt.chl.1 and was found in gene TraesCS7D02G040300 coding for the multi-protein family of sulfotransferase involved in postembryonic root development. In the same way, the QTLs namely. *Q*.*iari.dt.chl.4*, *Q*.*iari.dt.chl.5*, *Q.iari.dt.chl.6,* and *Q*.*iari.dt.chl.8* linked to SNPs AX94597699, AX95241386, AX94702861, and AX95230097 were harbouring the genes TraesCS6B02G056100, TraesCS6A02G037800, TraesCS6D02G046800, and TraesCS6D02G047400 coding for RNA-binding domain S1, ribosomal small subunit biogenesis (cleavage involved in rRNA processing), F-box-like domain superfamily and chloroplast rRNA processing, respectively.

**TABLE 4:** List of putative candidate genes and their functions.

Trait	Treatment	QTL	SNP ID	Chr	Traes ID	Position (bp)	Function	References
TLA	NLP	*Q.iari.dt.tla.1*	AX94470386	7B	TraesCS7B02G382300	648,103,932–648,109,965	ATPase-coupled cation transmembrane transporter activity	Sondergaard et al. (2004)
TLA	NLP	*Q.iari.dt.tla.2*	AX94765690	1D	TraesCS1D02G075500	57,476,559–57,477,974	Basic region/leucine zipper protein, positive regulation of transcription	Lastdrager et al. (2014), Zhang et al. (2020)
TLA	LP	*Q.iari.dt.tla.3*	AX94770913	6A	TraesCS6A02G406500	611,805,720–611,808,712	Ribosomal protein L9	Qi et al. (2019)
CHL	NLP	*Q.iari.dt.chl.1*	AX94832883	7D	TraesCS7D02G040300	20,397,341–20,399,320	Sulfotransferase involved in postembryonic root development	Zhou et al. (2010)
CHL	LP	*Q.iari.dt.chl.4*	AX94597699	6B	TraesCS6B02G056100	36,204,996–36,210,542	RNA-binding domain, S1	Young and Karbstein (2011)
CHL	LP	*Q.iari.dt.chl.5*	AX95241386	6A	TraesCS6A02G037800	18,704,699–18,708,252	Ribosomal small subunit biogenesis	Han et al. (2015)
CHL	LP	*Q.iari.dt.chl.6*	AX94702861	6D	TraesCS6D02G046800	21,050,249–21,055,169	F-box-like domain superfamily	Pérez-Torres et al. (2008)
CHL	LP	*Q.iari.dt.chl.8*	AX95230097	6D	TraesCS6D02G047400	21,980,166–21,985,295	chloroplast rRNA processing	Han et al. (2015)
SDW	NLP	*Q.iari.dt.sdw.4*	AX94503640	6D	TraesCS6D02G193000	267,200,426–267,202,351	Elongation factor Tu, translational elongation	Harvey *et al.* (2019)
SDW	NLP	*Q.iari.dt.sdw.5*	AX94812403	5A	TraesCS5A02G023600	18,795,384–18,797,642	Glycosyltransferase activity	Shi et al. (2020)
SDW	NLP	*Q.iari.dt.sdw.6*	AX94635019	4A	TraesCS4A02G307900	601,364,145–601,368,127	polysaccharide catabolic process	Yuan *et al.* (2019)
SDW	LP	*Q.iari.dt.sdw.9*	AX95081347	2D	TraesCS2D02G126300	73,425,329–73,426,739	Bulb-type lectin domain superfamily involved in plant development, stress response during germination and lateral root development	Vaid et al. (2013), Cheng et al. (2013), Deb et al. (2014)
SDW	LP	*Q.iari.dt.sdw.12*	AX94544797	1D	TraesCS1D02G218300	305,111,236–305,114,814	Phospho-2-dehydro-3-deoxyheptonate aldolase	Entus et al. (2002)
SDW	LP	*Q.iari.dt.sdw.15*	AX94939596	1D	TraesCS1D02G058900	38,780,587–38,789,555	Protein phosphorylation	Li *et al.* (2020)
SDW	LP	*Q.iari.dt.sdw.16*	AX95113278	5D	TraesCS5D02G496600	527,153,578–527,156,833	WAT1-related protein	Ranocha et al. (2013)
RSR	NLP	*Q.iari.dt.rsr.2*	AX94601118	2D	TraesCS2D02G190700	134,790,880–134,792,691	Protein dephosphorylation	Ma et al. (2021a)
RSR	NLP	*Q.iari.dt.rsr.6*	AX95190609	7A	TraesCS7A02G374700	547,568,878–547,574,155	Acetylglucosaminyltransferase activity	Yoo et al. (2021)
RSR	LP	*Q.iari.dt.rsr.12*	AX94944176	4D	TraesCS4D02G094500	69,846,691–69,850,833	Aldehyde dehydrogenase	Tola et al. (2020)
RSR	LP	*Q.iari.dt.rsr.13*	AX94460476	2D	TraesCS2D02G435000	545,937,573–545,939,217	Calcium-dependent protein binding	Reddy and Reddy, (2004)
TDW	NLP	*Q.iari.dt.tdw.4*	AX94572741	4B	TraesCS4B02G000900	621,192–623,232	ATG8-interacting protein	Michaeli *et al.* (2014)
TDW	LP	*Q.iari.dt.tdw.6*	AX94621027	7A	TraesCS7A02G383000	558,079,939–558,082,558	protein phosphorylation	Li et al. (2020)
TDW	LP	*Q.iari.dt.tdw.7*	AX94734828	7D	TraesCS7D02G243800	210,392,267–210,399,523	negative regulation of mRNA polyadenylation	Hunt (2012)
TDW	LP	*Q.iari.dt.tdw.9*	AX94426211	5B	TraesCS5B02G271700	457,128,028–457,130,661	Protein Iojap, chloroplastic	Carey (2016)
TDW	LP	*Q.iari.dt.tdw.10*	AX94884567	2A	TraesCS2A02G556400	760,619,027–760,620,516	Methyltransferase activity	Mishra et al. (2019)
TPC	NLP	*Q.iari.dt.tpc.1*	AX94905933	7A	TraesCS7A02G110500	67,671,323–67,674,222	F-box component of the SKP-Cullin-F box (SCF) E3 ubiquitin ligase complex, Strigolactone signal perception.	R. Liu et al. (2021)
TPC	LP	*Q.iari.dt.tpc.4*	AX94978370	3A	TraesCS3A02G298600	532,846,752–532,851,697	Integral component of membrane	Cvrcková (2000)
TPU	LP	*Q.iari.dt.tpu.3*	AX94713349	3A	TraesCS3A02G288900	517,074,705–517,080,156	Zinc Ion Binding	Cabot *et al.* (2019)

*Excluding common QTLs.

The SNP AX94514240 linked to the QTL *Q*.*iari.dt.sdw.1* was found in gene TraesCS1D02G029900 coding for leucine-rich repeat domain superfamily, NB ARC, P-loop containing nucleoside triphosphate hydrolase and in our study this gene has been found to play role in the expression in several traits like SDW, TDW, and TPU. The QTLs namely *Q*.*iari.dt.sdw.4*, *Q*.*iari.dt.sdw.5*, *Q.iari.dt.sdw.6,* and *Q*.*iari.dt.sdw.9* linked to SNPs AX94503640, AX94812403, AX94635019, and AX95081347 were having the genes TraesCS6D02G193000, TraesCS5A02G023600, TraesCS4A02G307900 and TraesCS2D02G126300 coding for elongation factor Tu and translational elongation, glycosyltransferase activity, polysaccharide catabolic process and bulb-type lectin domain superfamily (LecRLKs play important roles in plant development and stress responses, respectively). The protein coded by these genes plays an important role in seed germination and lateral root development. Other SNPs AX94544797, AX94939596, and AX95113278 and their associated QTLs *Q.iari.dt.sdw.12*, *Q*.*iari.dt.sdw.15,* and *Q*.*iari.dt.sdw.16* are localised in genes TraesCS1D02G218300, TraesCS1D02G058900, and TraesCS5D02G496600 coding for Phospho-2-dihydro-3-dioxyheptonate aldolase, protein phosphorylation and WAT1-related protein (facilitates auxin export), respectively.

Incidentally, the QTLs namely *Q*.*iari.dt.sdw.14* and *Q*.*iari.dt.sdw.20* linked to SNPs AX94626370 and AX94456805 and associated with SDW and TDW carried the genes TraesCS7B02G149200 and TraesCS2D02G584900 coding for Heat shock protein 90, cellular response to heat, protein stabilization, and Tubby-like F-box protein. The SNP AX94475513 linked with *Q*.*iari.dt.rsr.1* and associated with RSR and TDW has localized in gene TraesCS6A02G095100 coding for F-box-like domain superfamily, leucine-rich repeat domain superfamily, and leucine-rich repeat 2. The four QTLs associated with RSR viz., *Q*.*iari.dt.rsr.2*, *Q*.*iari.dt.rsr.6*, *Q*.*iari.dt.rsr.12,* and *Q*.*iari.dt.rsr.13* are located in genes coding for protein dephosphorylation, acetylglucosaminyltransferase activity, aldehyde dehydrogenase and calcium-dependent protein binding. Similarly, six QTLs *Q*.*iari.dt.tdw.4*, *Q*.*iari.dt.tdw.6*, *Q*.*iari.dt.tdw.7*, *Q*.*iari.dt.tdw.9*, *Q*.*iari.dt.tdw.10,* and *Q*.*iari.dt.tdw.14* are localized in genes TraesCS4B02G000900, TraesCS7A02G383000, TraesCS7D02G243800, TraesCS5B02G271700, TraesCS2A02G556400, and TraesCS7B02G149200 coding for ATG8-interacting protein, protein phosphorylation, negative regulation of mRNA polyadenylation, protein Iojap, chloroplastic, methyltransferase activity, and heat shock protein 90, respectively.

The QTLs namely *Q*.*iari.dt.tpc.1* and *Q*.*iari.dt.tpc.4* linked to SNPs AX94905933 and AX94978370 were harboring the genes TraesCS7A02G110500 and TraesCS3A02G298600 coding for the F-box component of the skp-cullin-f box (SCF) E3 ubiquitin ligase complex (Strigolactone (SL) signal perception, the unidirectional movement of auxin in the stem from tip to base basipetal) and integral component of membrane. *Q*.*iari.dt.tpc.2* QTL associated with traits TPC and PUtE was having the gene TraesCS3D02G267000 coding for glycerol lipid biosynthetic process, diacylglycerol O-acyltransferase activity. The *Q*.*iari.dt.tpu.3* was having the gene TraesCS3A02G288900 coding for Zinc ion binding.

### Putative candidate genes against wheat gene expression atlas

**FIGURE 7:**
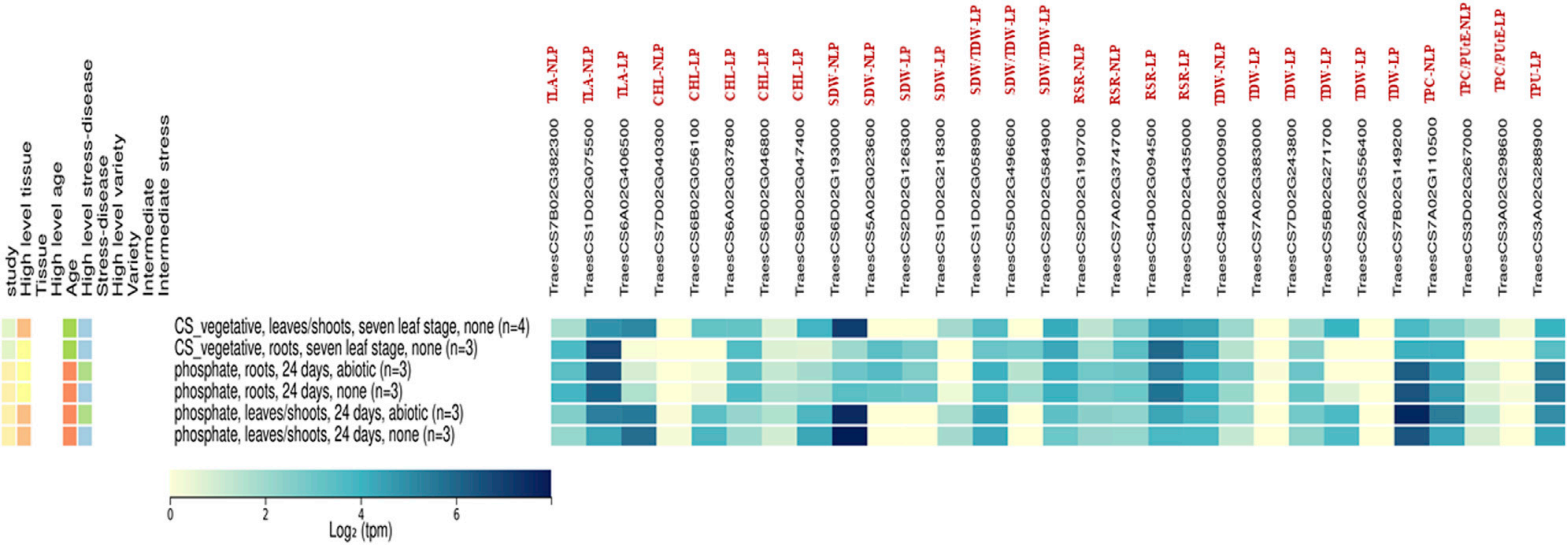
The expression analysis for identified Putative Candidate genes in Non-limiting and Limiting conditions by using the gene expression atlas.

Using the publicly available global gene expression atlas of wheat, the identified putative genes for P-responsive traits in our study were analysed against the gene expression atlas of wheat targeting the leaf and root traits under normal and phosphate deprivation conditions, to know the reliable expression of these genes (Borrill et al., 2016; Ramírez-González et al., 2018). Most of the identified genes were having the moderate to high consecutive expression in leaves and roots over normal and phosphate-deprivation conditions (Figure 7). These genes are TraesCS7B02G382300 and TraesCS1D02G075500 for TLA-NLP; TraesCS6A02G037800 for Chl-LP; TraesCS1D02G058900 and TraesCS2D02G584900 for SDW/TDW-LP; TraesCS2D02G190700, TraesCS7A02G374700, TraesCS4D02G094500 and TraesCS2D02G435000 for RSR-LP; TraesCS4B02G000900; TraesCS7A02G383000; TraesCS7D02G243800 and TraesCS7B02G149200 for TDW-LP; TraesCS7A02G110500 for TPC-NLP; TraesCS3D02G267000 for TPC/PUtE-NLP and TraesCS3A02G288900 for TPU-LP, which are consecutively expressed genes, except the non-expressed genes like TraesCS7D02G040300, TraesCS7A02G383000, TraesCS2A02G556400, and TraesCS3A02G298600. The other genes like TraesCS6A02G406500 for TLA-LP; TraesCS6B02G056100 and TraesCS6D02G047400 for Chl-LP, TraesCS6D02G193000 for SDW-LP; TraesCS1D02G218300 for SDW-LP and TraesCS5B02G271700 for TDW-LP were expressed in leaves and shoot of the wheat plants under normal and phosphorus deprivation conditions which coincides with our finding. The genes, TraesCS5A02G023600 for SDW-NLP; TraesCS2D02G126300 for SDW-LP; and TraesCS5D02G496600 for SDW/TDW-LP were expressed in root tissues under normal and phosphorus deprivation conditions.

## Discussion

To sustain wheat production globally through breeding intervention is the target of most breeding programs, primarily because wheat is the most important source of food and energy. The challenges to meeting this target are compounded by quickly depleting natural resources due to unsustainable management practices, declining soil health, and changing climatic conditions (Yadav et al., 2010)**.** Intensive cropping with no concern for resource use efficiency—such as is presently the case with phosphorus fertilizers—cannot be sustained for a very long period (Syers, Johnston, and Curtin, 2008; Lott et al., 2011). Under intensive cropping systems, increasing phosphorus efficiency has long been a target to sustain food production (Shenoy and Kalagudi, 2005; Schröder et al., 2011)**.** The residual soil phosphorus from past intense fertilization though contributes considerably to future crop output but with a large lag period as most of the applied phosphorus stays in the soil in the absence of larger uptake and efficient utilization (Sattari et al., 2012). As a result, improved plant capacity to utilize phosphorus effectively will be highly beneficial to crop output. The declining availability of rock phosphate as a source of phosphorus fertilizer and growing awareness about the negative consequences on the environment has piqued interest in improving plant phosphorus uptake and use efficiency (van de Wiel et al., 2016).

The results showed an increase in Chl, RSR, and PUtE and a decrease in TLA, SDW, TDW, TPC, and TPU under P-limited conditions. Improved phosphorus scavenging and uptake (phosphorus acquisition efficiency, PAE) achieved through better RSR with more economical and better utilization in the plant (phosphorus utilization efficiency, PUtE) as indicated in the present study can both improve phosphorus use efficiency (Wang, Shen, and Liao, 2010; Rose and Wissuwa, 2012; Veneklaas et al., 2012; van de Wiel et al., 2016). Correlation studies help to detect the relation between the traits, with respect to a specific treatment. Leaf area plays the most important role in carbon assimilation and therefore, was positively correlated with SDW, TDW, TPC, and TPU. The substantial contribution of TDW, phosphorus concentration, and total phosphorus uptake towards PUE was reported in rice (Wissuwa and Ae, 2001) and wheat (Valizadeh, Rengel, and Rate, 2002). However, better cell expansion to achieve optimum leaf area requires sufficient P in various plant parts, assimilation of which in fact depends upon better root proliferation and higher RSR. RSR exhibited considerably better expression in LP condition. In crop plants, non-availability of P predominantly stimulates root growth as opposed to shoot growth (Lambers et al., 2011). Chl was observed to have weak-to-no association with most of the other traits, but its expression manifests in LP conditions. Plants turn dark green in color when P deprivation is more severe (Hoppo, Elliott, and Reuter, 1999). Respiration and photosynthesis can be slowed down under phosphorus-deprived conditions (Glass, Beaton, and Bomke, 1980), but if respiration is slowed down more than photosynthesis, carbohydrates will be deposited, resulting in dark green leaves.

Principle component analysis clearly shows the importance of chlorophyll content, RSR, and PUtE under P-limited conditions whereas TPU, TPC, TLA, and TDW under nonlimiting P achieve higher yield. Plants have evolved highly specialized adaptive mechanisms through morphological, physiological, and molecular modifications such as an increased root/shoot ratio, an increase in the number of root hairs, association with arbuscular mycorrhizal fungi (AMF), synthesis and release of phosphatases and organic acids, and enhanced expression of phosphatases, to optimize access to soil phosphorus under limited phosphorus (Péret et al., 2011). The length of the vector (distance from the origin) explains the variation contributed by each trait. Along with mean values and correlation, PCA explains the traits TPU, TPC, TLA, SDW, and TDW are highly correlated and explains the large amount of variation contributed by these traits. The significant crossover interaction between traits across environments (i.e > 90°) helps to identify the traits to be useful for the selection/improvement of genotypes for a specific environment (Yan and Tinker, 2006). In the present study HDCSW18, a wheat variety of very high yield potential and specifically bred for conservation agriculture conditions, exhibits very high phosphorus limitation tolerance largely because of its strong root traits. This genotype because of its strong RSA traits (Dharmateja et al., 2021) has the inherent ability to explore even the deeper layers of soil. Our study clearly shows that better P uptake though, largely depends upon root traits, and is essential for better ground coverage and C assimilation but can improve PUE up to a limited extent because of the limitation imposed by PUtE. It also vouches for a separate breeding programme for both sets of conditions.

For mapping, a panel of 158 wheat genotypes was used, including advanced breeding lines, obsolete varieties, and recently released varieties. The population structure revealed by STRUCTURE analysis infers two major sub-populations (Figure 4A). The presence of two major sub-populations, was further confirmed with the dendrogram and constellation plot. The genotypes in a group shared alleles descended from common parents leading to genetic relatedness among the genotypes. The genotypes are mostly grouped based on pedigree lineage, and evolutionary and geographical origin (Gorafi et al., 2018; Tomar et al., 2021). In view of genetic relatedness, we have adopted the FarmCPU, BLINK, and MLM with population structure and kinship relatedness matrix in association analysis to avoid spurious false positives. The genomic regions responsible for better trait expression both under abundant and deficit P conditions were identified through GWAS. In total 38 QTLs under NPL and 45 QTLs under LP were associated with various traits in the present study. Four QTLs for TLA, eight for Chl, twenty for SDW, seventeen for RSR, eighteen for TDW, five for TPC, seven for TPU, and four QTLs for PUtE were found to be associated in both conditions. In developing countries like India, the major focus of the breeders throughout the 20th century was to achieve higher yields with almost nil or negligible effort on the development of nutrient-efficient genotypes. However, with the faster depletion of natural resources for P and stronger dependence on imports, it becomes inevitable to focus on the development of phosphorus efficient genotypes in wheat. In contrast, breeding phosphorus-efficient wheat genotypes has received a lot of attention (Davies et al., 2002; Wang et al., 2005) in the developed world where genotypic differences in phosphorus acquisition efficiency and phosphorus utilization efficiency for wheat have been frequently reported (Batten, 1993; Manske and Vlek, 2002; Wang et al., 2005; Gunes et al., 2006). The shoot traits are mostly associated and their biological process are also presumed to coordinate with their expression. Strong pleiotropic gene action or tight linkage between the genes results in strong correlation between the traits. Five QTLs in NLP, three QTLs in LP, and one in both treatments were detected for multiple traits. The loci affecting multiple traits should be a potential marker for marker-assisted selection for varietal improvement (Chebib and Guillaume, 2021).

### Putative candidate gene functions of identified QTLs

Studying the annotated genomic region in wheat enabled us to identify the genes within the associated SNPs/identified QTLs. The identified putative candidate gene for *Q.iari.dt.tla.1* on 7B for TLA is reported to be responsible for ATPase-coupled cation transmembrane transporter activity. H + -ATPase had a role in nutrient uptake in the root and translocation of these nutrients to the shoots (Sondergaard, Schulz, and Palmgren, 2004). Similarly, *Q.iari.dt.tla.2* for TLA was linked with the gene responsible for basic leucine zipper protein, which is reported to have a role in the positive regulation of transcription. Plants regulate various physiological processes through a regulatory network of transcription factors. Under nutrient starvation, the conserved sucrose-non-fermenting-1-related protein kinase-1 (SnRK1) mediates the phosphorylation of S1-bZIPs (basic region/leucine zipper) to regulate plant growth and development (Lastdrager et al., 2014). SnRK1 also play a major role in low energy syndrome response under stress condition (Zhang et al., 2020). Mitochondrial ribosomal protein L9, crucial for cells’ survival and kernel development regulation (Qi et al., 2019), was linked with *Q.iari.dt.tla.3*.

*Q.iari.dt.chl.1* on chromosome 7D is linked to the gene responsible for sulfotransferase. In *Arabidopsis thaliana*, tyrosyl protein sulfotransferase (TPST) was found to act in the auxin/plethora pathway to maintain the stem cell niche of the roots (Zhou et al., 2010). *Q.iari.dt.chl.4* is associated with the RNA-binding domain, S1. As distinct RNA-binding domains (RBDs) are very limited in number, they often combine with multiple RNA-binding motifs for higher affinity and target selectivity. S1 domains binding RNA specifically and non-specifically with high affinity indicate its importance in Rrp5 (ribosomal assembly factor), pre-rRNA complex (Young and Karbstein, 2011). *Q.iari.dt.chl.6* was an ensemble with a gene F-box-like domain superfamily. F-box containing highly conserved motif and their association with cellular degradation with other interacting domains have a robust adaptive role under biotic and abiotic stress conditions, including low-P stress in crops (Pérez-Torres et al., 2008). *Q.iari.dt.chl.8* on chromosome 6D is associated with the gene responsible for chloroplast rRNA processing. The rRNA processing is critical for chloroplast biogenesis and photosynthetic activity resulting in the normal growth of Arabidopsis (Han et al., 2015).
*Q.iari.dt.sdw.1* is present on chromosome 1D and associated with nucleotide-binding sites -leucine-rich repeat domain responsible for plant proteins’ key role in host-pathogen interaction (Flor, 1956; Hammond-Kosack and Jones, 1997). Similarly, *Q.iari.dt.sdw.5* associated with gene glycosyltransferase OsUGT90A1 on chromosome 5A helps in protecting the plasma membrane during stress (Shi et al., 2020). *Q.iari.dt.sdw.9* present on chromosome 2D is linked to the bulb-type lectin domain superfamily. Lectin receptor-like kinase (LecRLKs) is reported to play essential roles in plant development and stress responses (Vaid, Macovei, and Tuteja, 2013) besides their involvement in germination processes (Cheng et al., 2013) and lateral root development (Deb et al., 2014). *Q.iari.dt.sdw.14* is associated with heat shock protein 90 which plays an important role in plant adaptation under different environmental conditions (Wang et al., 2011; Kumar et al., 2020). *Q.iari.dt.sdw.15* is associated with gene-responsible protein phosphorylation, which is an integral part of abiotic stress-responsive pathways including phytohormones and ion homeostasis. Auxin hormone plays a very important role in plant adaptation response and the QTL *Q.iari.dt.sdw.16* is associated with a candidate gene responsible for WALLS ARE THIN1 (WAT1), a plant-specific protein that facilitates auxin export from vacuoles (Ranocha et al., 2013). Tubby-like proteins (TLPs) are present in all eukaryotic species (Liu, 2008), including wheat (Hong, Kim, and Seo, 2016). *Q.iari.dt.sdw.20* on chromosome 2D was found associated with a Tubby-like F-box protein-producing gene reported to have a role in adaptation response though with some indistinct mechanism.
QTL *Q.iari.dt.rsr.1* was found to be putatively associated with the gene F-box-like domain superfamily. F-box genes in plants control many important processes including embryogenesis, hormonal responses, seedling development, floral organogenesis, senescence, and pathogen resistance (Lechner et al., 2006). *Q.iari.dt.rsr.2* on chr2D was found putatively associated with gene coding protein dephosphorylation. Plants regulate protein through phosphorylation and dephosphorylation during their response to biotic and abiotic stresses (Ma et al., 2021b). Gene regulating acetylglucosaminyltransferase was found putatively associated with *Q.iari.dt.rsr.6*, the role in various developmental processes under stress conditions has been established in Arabidopsis (Yoo et al., 2021). *Q.iari.dt.rsr.12* was associated with gene-regulating Aldehyde dehydrogenase production, which oxidizes excessive endogenous and exogenous aliphatic and aromatic aldehyde molecules into corresponding carboxylic acids (Tola et al., 2020). *Q.iari.dt.rsr.13* on chromosome 2D is putatively associated with the calcium-dependent protein binding gene. Calcium ions are a messenger for physiological responses to various developmental signals (Reddy and Reddy, 2004).
During the energy deprivation-induced by limiting nutrient supply or other biotic and abiotic stresses, including toxicity, plants have evolved the autophagy process to counter the negative outcome. *Q.iari.dt.tdw.4* links were found putatively associated with ATG8-interacting protein. Similarly, phosphorylation is an important means through which plants regulate post-translational gene expression. Q*.iari.dt.tdw.6* was found to be associated with genes responsible for phosphorylation. *Q.iari.dt.tdw.7* was found to be associated with gene-regulating negative mRNA polyadenylation. Plants also regulate gene expression quantitatively and qualitatively through mRNA polyadenylation (Hunt, 2012). *Q.iari.dt.tdw.9* is an ensemble with a gene responsible for IOJAP protein localized in the chloroplast in Arabidopsis and found to have a role in adaptation to cold stress. *Q.iari.dt.tdw.10* is associated with gene methyltransferase conferring tolerance to salinity stress in Arabidopsis (Mishra et al., 2019). *Q.iari.dt.tpc.1* is related to the gene F-box component of the SKP-Cullin-F box (SCF) E3 ubiquitin ligase complex. The complex is involved in various growth, flower development, and other physiological processes in wheat (Liu et al., 2021).

As earlier discussed, some of the QTLs were associated with more than one trait and which harbors the putative candidate genes. The *Q.iari.dt.sdw.1* associated with SDW, TDW, and TPU under NLP, and harboring the TraesCS1D02G029900 which code for Leucine-rich repeat domain superfamily, NB ARC, P-loop containing nucleoside triphosphate hydrolase responsible for plant proteins’ key role in host-pathogen interaction (Flor, 1956; Hammond-Kosack and Jones, 1997) (Table 3). Another two more QTLs viz. *Q*.*iari.dt.sdw.14* and *Q*.*iari.dt.sdw.20* were associated with SDW and TDW, and harbouring the TraesCS7B02G149200 and TraesCS2D02G584900, respectively. The putative candidate genes, TraesCS7B02G149200 codes for heat shock protein 90 which plays an important role in plant adaptation under different environmental conditions (Wang et al., 2011; Kumar et al., 2020), while, TraesCS2D02G584900 codes for Tubby-like F-box protein involved in various physiological activities (Hong, Kim, and Seo, 2016). Likewise, QTL named *Q*.*iari.dt.rsr.1* was associated with RSR and TDW under both conditions, and harbouring the TraesCS6A02G095100 responsible for F-box-like domain super family, FBD domain, Leucine-rich repeat domain superfamily influences the embryogenesis, hormonal responses, seedling development, floral organogenesis, senescence, and pathogen resistance (Lechner et al., 2006). The QTL, *Q*.*iari.dt.tpc.2* associated with PUtE and TPU, was harbouring the TraesCS3D02G267000 which codes for Glycerolipid biosynthetic process, diacylglycerol O-acyltransferase activity responsible for lipid metabolism (Sanjaya et al., 2013).

In the Wheat Gene Expression Atlas data, several transcripts of different wheat tissues with various ID contents exhibit differential gene expression (Borrill, Ramirez-Gonzalez, and Uauy, 2016). The several genes expressed in leaves, roots, and shoots of the wheat plants under normal and phosphorus-deprived conditions which indicate the potential tissue-specific roles that these genes play in phosphorus stress. Many QTLs explaining the significant variation for many root traits relevant for PUE in the present study are co-localized in the chromosome regions harbouring essential pertinent genes for stress response and growth development processes in crop plants. Isolation, cloning, and verifying their role in PUE may pave the way for developing stable molecular markers in the crop improvement programme. The expression pattern of identified putative genes against the gene expression atlas indicates that the identified genes in this study have relevance, which can be converted into PCR-based primers for marker-assisted selection.

## Conclusion

Identifying regions on the chromosome in the form of QTLs to explain the phenotypic variation for the various breeding traits is an important tool to improve breeding programme efficiency. The identified genomic loci (83 loci across the models and treatments) in the present study explain the significant variation in phosphorus uptake and utilization or its associated root and shoot traits. Their connotation with putative functions or proteins can lead to the validation of gene(s) underlying these loci. The discovered common QTLs controlling several phenotypes may serve as candidate markers for marker-assisted breeding. However, the functional markers need to be validated in a separate independent panel with different genetic backgrounds of wheat genotypes. The proteins encoded by the identified genes are involved in many developmental processes, particularly stress responses. Therefore, there is a need to investigate the functions, activation, deactivation, or changes in the expression rate of these genes in the different developmental stages, to know how these genes enhance the efficiency of a few genotypes under P deficient or P surplus conditions.

Further research into the genic regions of associated SNPs at the transcriptional level is needed to determine the trustworthy source of efficiency imparted in the genotypes studied, which will aid in the identification of distinct developmental pathways. Given the rising cost and relevance of phosphorus as an agricultural input, Crop improvement, PUE is an intrinsically worthwhile objective. However, a PUE-focused breeding programme will compete with other breeding goals like disease resistance and climate change adaptability. The discovery of QTL allows for the creation of trait-relevant markers for marker-assisted or genomic selection methods. Overall, the vast diversity of the genetic resources used in this study will help develop new cultivars of wheat with higher PUE by genomic-assisted breeding.

## Data Availability

The original contributions presented in the study are publicly available. This data can be found here: https://doi.org/10.6084/m9.figshare.20298006.v1.
