# Supplementary material for: Genome-wide association studies reveal putative QTLs for physiological traits under contrasting phosphorous conditions in wheat (Triticum aestivum L.)
Source: Front Genet. 2022 Nov 11;13:984720. doi: 10.3389/fgene.2022.984720 (PMC9691895; doi:10.3389/fgene.2022.984720)

Supplementary Figure 1: Manhattan plots and Q-Q plots (Blink: NLP-1A, LP-1B) (MLM: NLP-1C, LP-1D) for Phosphorus use efficiency traits. TLA, total leaf area; Chl, chlorophyll content; SDW, shoot dry weight; RSR, root:shoot ratio; TDW, total dry weight; TPC, total phosphorus content; TPU, total phosphorus uptake; PUtE, phosphorus utilization efficiency;

1A


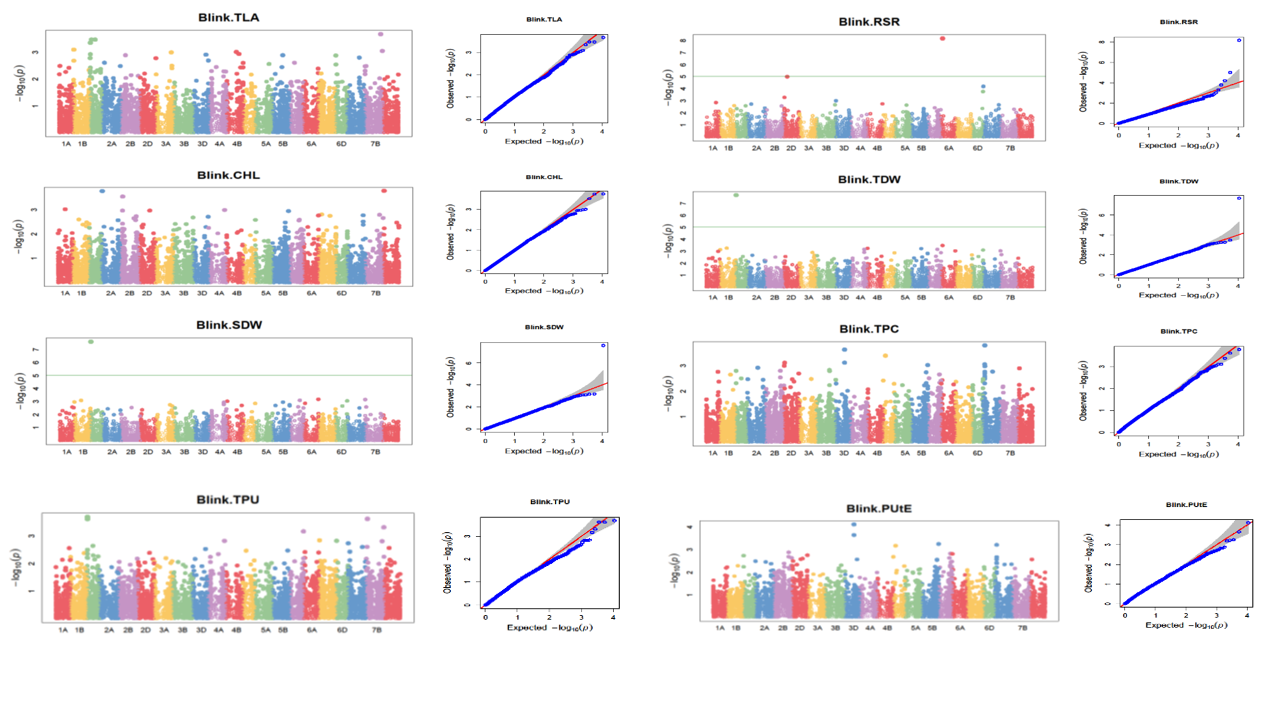


1B


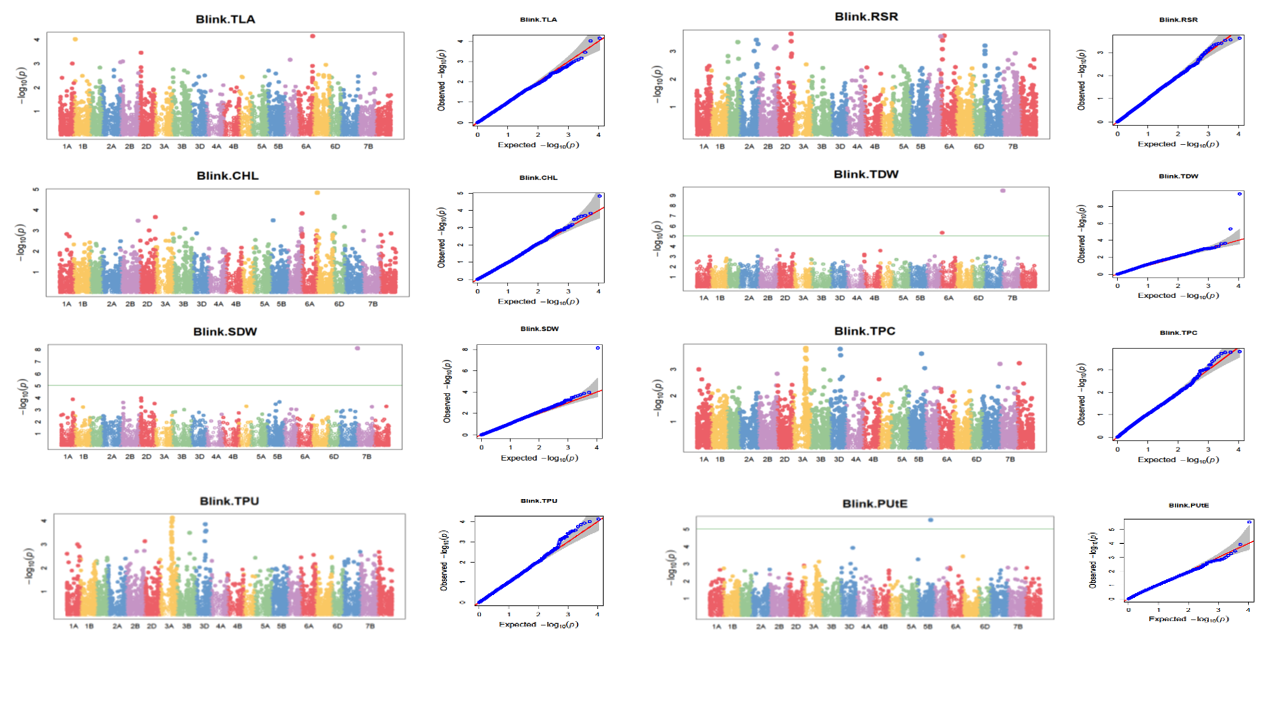


1C


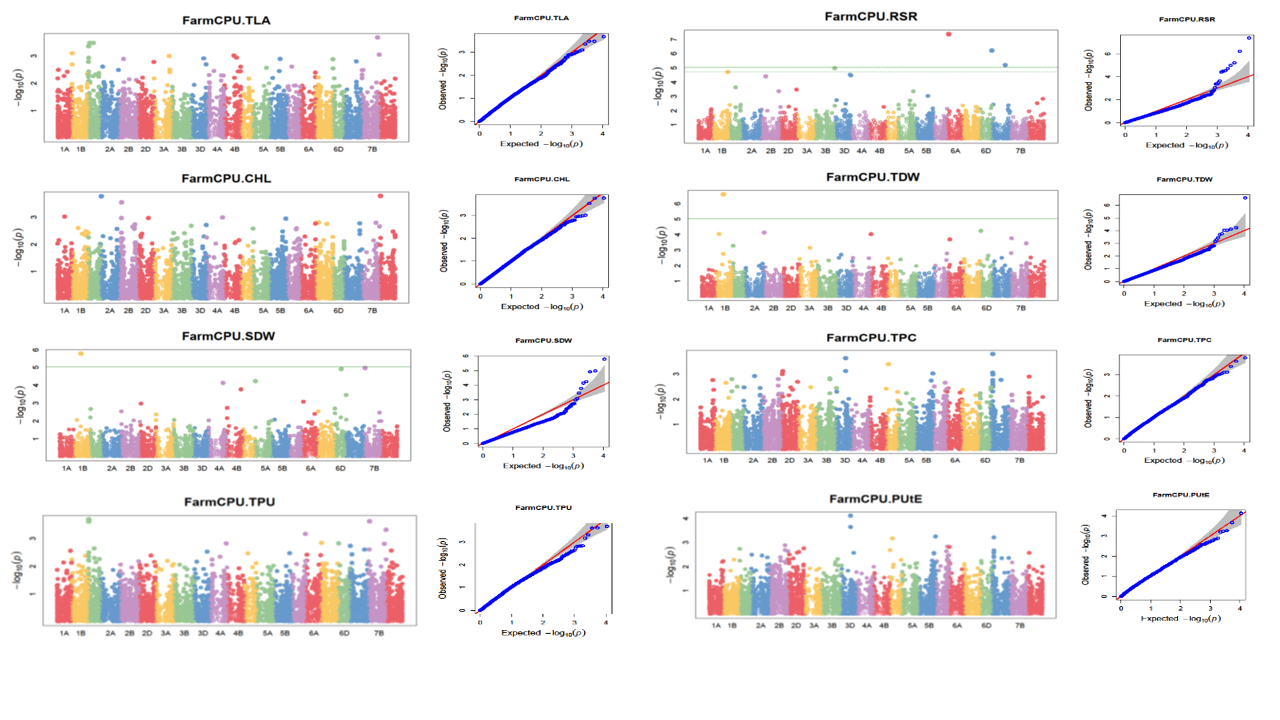


1D


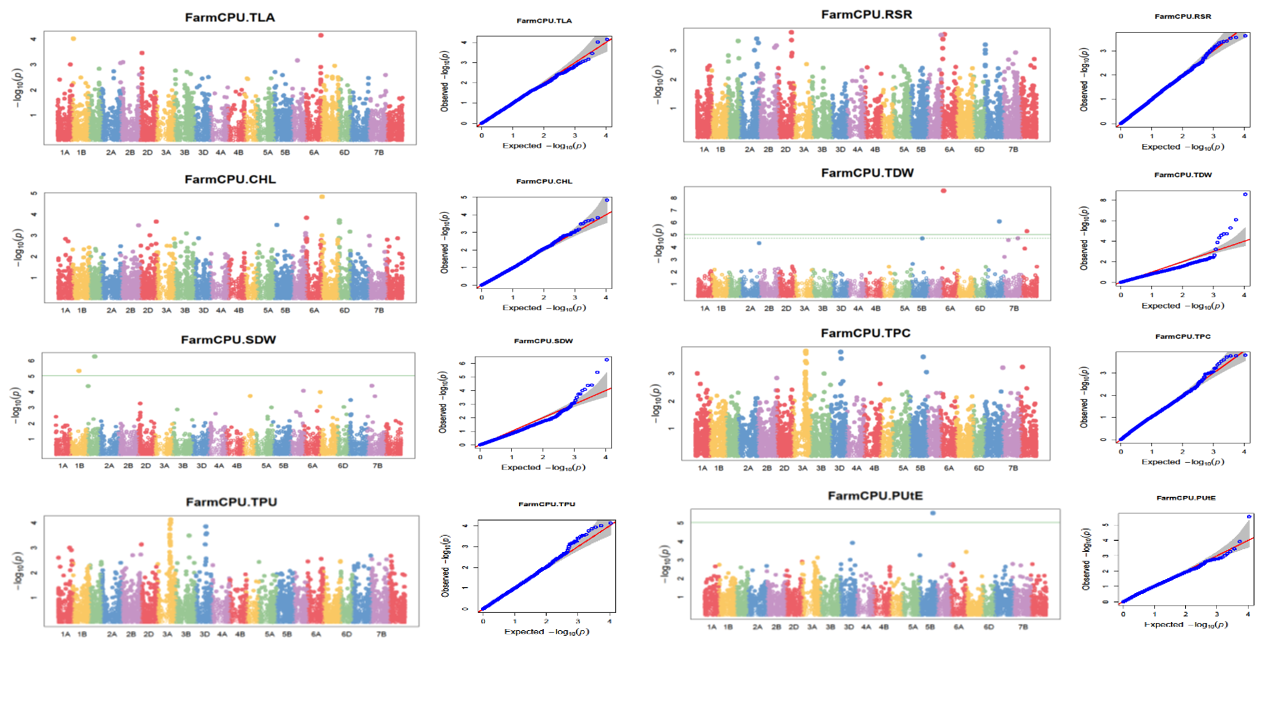

Supplement: Supplementary file 1 [file Table1.DOCX]
