# Supplementary material for: Genome-wide association studies reveal putative QTLs for physiological traits under contrasting phosphorous conditions in wheat (Triticum aestivum L.)
Source: Front Genet. 2022 Nov 11;13:984720. doi: 10.3389/fgene.2022.984720 (PMC9691895; doi:10.3389/fgene.2022.984720)
Supplement: Supplementary file 2 [file DataSheet1.docx]

**Supplementary table 1: parentage of 158 genotypes used in the study**

| **Genotype** | **Pedigree** | **Varieties/ Breeding lines** |
| --- | --- | --- |
| BW60 | VL332/Wei-132/VL829//VmI-39-II | Breeding line |
| BW63 | HD3059/HD3054 | Breeding line |
| BW44 | HD2953/HS365 | Breeding line |
| BW65 | 31ESWYT-135//HD2329/WR544/PBW343/NW3041 | Breeding line |
| BW69 | CSW2/Yr15 | Breeding line |
| BW96 | HD3115/PBW550 | Breeding line |
| BW99 | HD3226 | Variety |
| BW58 | SAWYT-326/HD2967 | Breeding line |
| BW51 | CL1705/HD2687 | Breeding line |
| BW66 | HD3226/HD3086//HDCSW18 | Breeding line |
| BW43 | HD3086 | Variety |
| BW68 | 31-ESWYT-132/CSW23 | Breeding line |
| BW11 | HD3086 | Mutant |
| BW88 | HD2967/RSP566 | Breeding line |
| BW20 | CL1591/CL1435//HD2967 | Breeding line |
| BW90 | IM-15/HD2967 | Breeding line |
| BW93 | 43IBWSN-1175 | Breeding line |
| BW37 | 43IBWSN-1175 | Breeding line |
| BW98 | CSW35/DBW17 | Breeding line |
| BW33 | CL264/CL1633//CNo-601 | Breeding line |
| BW48 | CL264/CL1633//CNo-601 | Breeding line |
| BW86 | HD2953/HS365 | Breeding line |
| BW1 | CSW2/Yr15 | Breeding line |
| BW165 | HD2967/CP196 | Breeding line |
| BW178 | IM-36/HD2967 | Breeding line |
| BW179 | VL849/NBP-39//HD2967 | Breeding line |
| BW180 | HD3086 | Mutant |
| BW144 | SAWYT-326/HD2967 | Breeding line |
| BW169 | CSW3/HD2932+Yr10 | Breeding line |
| BW163 | HD2329/HDK10//CBW38/WR544 | Breeding line |
| BW168 | HD2874/HD2967//43^rd^IBWSN-1087 | Breeding line |
| BW142 | HD2329/HD2285//43IBWSN-1182 | Breeding line |
| BW164 | 31ESWYT-132/CSW-17 | Breeding line |
| BW177 | 31ESWYT-135/CSW23 | Breeding line |
| BW146 | SAWYT-326/HD2967 | Breeding line |
| BW143 | CSW2/Yr15 | Breeding line |
| BW172 | SAWYT-326/HD2967 | Breeding line |
| BW154 | SAWYT-326/HD2967 | Breeding line |
| BW171 | CSW2/Yr15 | Breeding line |
| BW156 | HD2967//HD2887/HD2946//HD2733 | Breeding line |
| BW157 | HD2967 | Variety |
| BW158 | HD2967//HD2887/HD2946//HD2733 | Breeding line |
| BW176 | HD2967//HD2887/HD2946//HD2733 | Breeding line |
| BW175 | HD2967//HD2887/HD2946//HD2733 | Breeding line |
| BW167 | HD2967//HD2887/HD2946//HD2733 | Breeding line |
| BW155 | HD2967//HD2887/HD2946//HD2733 | Breeding line |
| BW145 | SAWYT-326/HD2967 | Breeding line |
| BW116 | CSW3/HD2932+Yr10 | Breeding line |
| BW104 | HD3117 | Variety |
| BW102 | CSW3/HD2932+Yr10 | Breeding line |
| BW147 | IM-15/HD2967 | Breeding line |
| BW141 | IM-15/HD2967 | Breeding line |
| BW166 | IM-15/HD2967 | Breeding line |
| BW101 | IM-15/HD2967 | Breeding line |
| BW153 | HD3226 | Mutant |
| BW109 | HD2967//HD2887/HD2946//HD2733 | Breeding line |
| BW110 | HD2877/DW343//WH542/3/HD2982//HD2967 | Breeding line |
| BW136 | CSW3/HD2932+Yr10 | Breeding line |
| BW106 | CSW3/HD2932+Yr10 | Breeding line |
| BW148 | HD3086 | Mutant |
| BW162 | CSW2/Yr15 | Breeding line |
| BW103 | CSW18/CSW1 | Breeding line |
| BW111 | CSW18/CSW1 | Breeding line |
| BW115 | CSW18/CSW1 | Breeding line |
| BW113 | CSW18/CSW1 | Breeding line |
| BW137 | CSW18/CSW1 | Breeding line |
| BW105 | HD2789/HD2891//HD2932 | Breeding line |
| BW133 | UP2425/Blend-b-I-II | Breeding line |
| BW126 | CSW01/DBW17 | Breeding line |
| BW151 | HD2967//HD2887/HD2946//HD2733 | Breeding line |
| BW140 | CSW88 | Breeding line |
| BW139 | HD2967//HD2887/HD2946//HD2733 | Breeding line |
| BW152 | HDCSW18 | Variety |
| BW150 | HD2967/CP196 | Breeding line |
| BW121 | IM-15/HD2967 | Breeding line |
| BW159 | 31stESWYT135/CSW23/18 | Breeding line |
| BW125 | 31^st^ESWYT-135//HD2329/WR544/PBW343/NW3041 | Breeding line |
| BW160 | 31ESWYT-138/CSW30 | Breeding line |
| BW127 | CSW90 | Breeding line |
| BW112 | HD2967//HDCSW16/HD3054 | Breeding line |
| BW118 | HD3226 | Mutant |
| BW134 | 18HRWYT214/18 HRWYT-229 | Breeding line |
| BW135 | 31ESWYT-135/CSW23 | Breeding line |
| BW120 | HD3086//HD3059 | Breeding line |
| BW138 | CSW90/HD3086 | Breeding line |
| BW114 | HD2784/HD3059 | Breeding line |
| BW183 | HD3117 | Mutant |
| BW107 | HD3086//HD2329 | Breeding line |
| BW117 | HD CSW18/HD2329 | Breeding line |
| BW129 | CSW18/HD2967 | Breeding line |
| BW50 | 31ESWYT-138/CSW30 | Breeding line |
| BW35 | 31-ESWYT-138/CSW30 | Breeding line |
| BW27 | IRSBWYT-19 | Breeding line |
| BW123 | HD2967/HDCSW16 | Breeding line |
| BW128 | HD3115/PBW550 | Breeding line |
| BW130 | PBW504/VL849//43^rd^IBWSN-1164 | Breeding line |
| BW25 | SAWYT-326/HD2967 | Breeding line |
| BW81 | CSW30/CSW34 | Breeding line |
| BW32 | CSW2/Yr15 | Breeding line |
| BW28 | CSW/Yr15 | Breeding line |
| BW6 | CSW2/Yr15 | Breeding line |
| BW57 | 31^st^ESWYT139//PBW343/PH 137//MC-11 | Breeding line |
| BW82 | 22^nd^HRWSN-2112 | Breeding line |
| BW13 | IM-15/HD2967 | Breeding line |
| BW7 | HD2789/HD2891//HD2932 | Breeding line |
| BW100 | HD3086 | Mutant |
| BW80 | HD2967//HD2887/HD2946//HD2733 | Breeding line |
| BW55 | 31ESWYT-135/CSW23 | Breeding line |
| BW10 | HD2967//HD2887/HD2946//HD2733 | Breeding line |
| BW2 | HD2967//HD2887/HD2946//HD2733 | Breeding line |
| BW9 | HD2967//HD2887/HD2946//HD2733 | Breeding line |
| BW54 | 31ESWYT-135//HD2329 | Breeding line |
| BW64 | C306//HI154/HD2967 | Breeding line |
| BW92 | CSW3/HD2932+Yr10 | Breeding line |
| BW74 | 18HRWYT214/18 HRWYT-229 | Breeding line |
| BW182 | C306/HD2329//HD2932/HD3059 | Breeding line |
| BW53 | 31-ESWYT-135//HD2329/WR544/PBW343/NW3041 | Breeding line |
| BW3 | HD2967//HD2887/HD2946//HD2733 | Breeding line |
| BW29 | CSW/Yr15 | Breeding line |
| BW83 | HD2967//HD2887/HD2946//HD2733 | Breeding line |
| BW75 | 31-ESWYT-147/3/HW5028//HD2432/DW2009 | Breeding line |
| BW73 | IBWSN//HD30/HD2824 | Breeding line |
| BW46 | VL616(2)Inqualab/Kundan | Breeding line |
| BW192 | HD 1962/E 4870/3/K 65/5/HD1553/4/UP262 | Breeding line |
| BW181 | CSW2/HDCSW18//HD 2784 | Breeding line |
| BW193 | PBW 343/CL 1538 | Breeding line |
| BW194 | Type 9/8B | Breeding line |
| BW195 | Grackle/HD 2894 | Breeding line |
| BW196 | KAUZ/STAR//HD2643 | Breeding line |
| BW197 | SEL. HETEROZYGOUS Line of Local MUNDIA | Breeding line |
| BW198 | KAUZ//ALTAR84/AOS/3/MILAN/KAUZ/4/HUITES | Breeding line |
| BW199 | KAUZ//ALTAR84/AOS/3/MILAN/KAUZ/4/HUITES | Breeding line |
| BW200 | S 308/ CHR//KAL | Breeding line |
| BW201 | ALD/COC//URES/HD2160M/HD2278 | Breeding line |
| BW184 | CSW109 | Breeding line |
| BW185 | CSW/Yr15 | Breeding line |
| BW186 | HD2967//HD2887/HD2946//HD2733 | Breeding line |
| BW97 | 31ESWYT-138/CSW23 | Breeding line |
| BW187 | HD2877/DW343//WH542/3/HD2982//HD2967 | Breeding line |
| BW188 | HD2967//HD2887/HD2946//HD2733 | Breeding line |
| BW189 | CL264/CL1633//CNo-601 | Breeding line |
| BW190 | SAWYT-326/HD2967 | Breeding line |
| BW191 | CL1705/HD2687 | Breeding line |
| BW202 | JUP /BJY”S”//URES | Breeding line |
| BW203 | DO/E518//SPP/NP114/3/WIS245'S' | Breeding line |
| BW174 | HD2967//HD2887/HD2946//HD2733 | Breeding line |
| BW131 | CSW25/CSW18//HD2967 | Breeding line |
| BW204 | PJ'S'/GB55 | Breeding line |
| BW205 | YT54/N10B//2*Y54 | Breeding line |
| BW206 | LR 64A /NAI 60 | Breeding line |
| BW207 | CMH79A.95/3*CNO79//RAJ3777 | Breeding line |
| BW208 | HW 2002/WR196 | Breeding line |
| BW209 | WH 594/RAJ 3858//W485 | Breeding line |
| BW91 | ICSISA-SBMNP-4-158 | Breeding line |
| BW59 | HD3086 | Mutant |
| BW210 | HD2733/HD2824//DW1278 | Breeding line |
| BW211 | PBW 343/HD2879 | Breeding line |
| BW22 | CSW114 | Breeding line |

*All the breeding lines/varieties are developed from IARI station, New Delhi

**Supplementary Table 2:** SNPs distribution across the wheat chromosomes.

| **Genome** | **Chromosome** | | | | | | | **Total** |
| --- | --- | --- | --- | --- | --- | --- | --- | --- |
|  | **1** | **2** | **3** | **4** | **5** | **6** | **7** |  |
| **A** | 500 | 587 | 457 | 352 | 525 | 412 | 576 | 3409 |
| **B** | 679 | 749 | 566 | 277 | 675 | 571 | 524 | 4041 |
| **D** | 639 | 707 | 501 | 180 | 500 | 348 | 475 | 3350 |

**Supplementary Table 3: List of Marker-Trait Associations (MTAs) detected for phosphorus use efficiency traits using three models: BLINK, FARM CPU, and MLM**

| **Trait** | **Treatment** | **QTL** | **SNP** | **Chr** | **Position** | **Model** | **P. Value** | **MAF** | **Effect** | **-LOG10 P VALUE** |
| --- | --- | --- | --- | --- | --- | --- | --- | --- | --- | --- |
| TLA | NLP | *Q.iari.dt.tla.1* | AX94470386 | 7B | 648106895 | BLINK | 0.000210 | 0.484 | NA | 3.7 |
|  |  |  |  |  |  | FARM CPU | 0.000210 | 0.484 | -6.770 | 3.7 |
|  |  |  |  |  |  | MLM | 0.0001994 | 0.484 | -6.206 | 3.7 |
|  |  | *Q.iari.dt.tla.2* | AX94765690 | 1D | 57476693 | BLINK | 0.0003347 | 0.085 | NA | 3.5 |
|  |  |  |  |  |  | FARM CPU | 0.0003347 | 0.085 | -11.552 | 3.5 |
|  | LP | *Q.iari.dt.tla.3* | AX94770913 | 6A | 611808086 | BLINK | 7.02E-05 | 0.120 | NA | 4.2 |
|  |  |  |  |  |  | FARM CPU | 7.02E-05 | 0.120 | -4.841 | 4.2 |
|  |  |  |  |  |  | MLM | 2.76E-05 | 0.120 | -5.028 | 4.6 |
|  |  | *Q.iari.dt.tla.4* | AX95190390 | 1B | 48689512 | BLINK | 9.48E-05 | 0.066 | NA | 4.0 |
|  |  |  |  |  |  | FARM CPU | 9.48E-05 | 0.066 | -5.361 | 4.0 |
|  |  |  |  |  |  | MLM | 5.28E-05 | 0.066 | -5.544 | 4.3 |
| Chl | NLP | *Q.iari.dt.chl.1* | AX94832883 | 7D | 20397481 | BLINK | 0.0001698 | 0.108 | NA | 3.8 |
|  |  |  |  |  |  | FARM CPU | 0.0001698 | 0.108 | 2.677 | 3.8 |
|  |  | *Q.iari.dt.chl.2* | AX94676652 | 2A | 44818131 | BLINK | 0.0001738 | 0.142 | NA | 3.8 |
|  |  |  |  |  |  | FARM CPU | 0.0001738 | 0.142 | 2.221 | 3.8 |
|  |  | *Q.iari.dt.chl.3* | AX95105278 | 2B | 104832888 | BLINK | 0.0002913 | 0.389 | NA | 3.5 |
|  |  |  |  |  |  | FARM CPU | 0.0002913 | 0.389 | -1.238 | 3.5 |
|  | LP | *Q.iari.dt.chl.4* | AX94597699 | 6B | 36206328 | BLINK | 1.455E-05 | 0.342 | NA | 4.8 |
|  |  |  |  |  |  | FARM CPU | 1.455E-05 | 0.342 | 1.714 | 4.8 |
|  |  |  |  |  |  | MLM | 1.808E-05 | 0.342 | 1.742 | 4.7 |
|  |  | *Q.iari.dt.chl.5* | AX95241386 | 6A | 18706523 | BLINK | 0.000145 | 0.367 | NA | 3.8 |
|  |  |  |  |  |  | FARM CPU | 0.000145 | 0.367 | 1.479 | 3.8 |
|  |  |  |  |  |  | MLM | 0.0001201 | 0.367 | 1.520 | 3.9 |
|  |  | *Q.iari.dt.chl.6* | AX94702861 | 6D | 21051029 | BLINK | 0.0001931 | 0.500 | NA | 3.7 |
|  |  |  |  |  |  | FARM CPU | 0.0001931 | 0.500 | -1.301 | 3.7 |
|  |  |  |  |  |  | MLM | 0.0002256 | 0.500 | -1.354 | 3.6 |
|  |  | *Q.iari.dt.chl.7* | AX94622481 | 2D | 645683597 | BLINK | 0.0002182 | 0.149 | NA | 3.7 |
|  |  |  |  |  |  | FARM CPU | 0.0002182 | 0.149 | -2.525 | 3.7 |
|  |  | *Q.iari.dt.chl.8* | AX95230097 | 6D | 21981934 | BLINK | 0.0002469 | 0.386 | NA | 3.6 |
|  |  |  |  |  |  | FARM CPU | 0.0002469 | 0.386 | 1.383 | 3.6 |
|  |  |  |  |  |  | MLM | 0.000173 | 0.386 | 1.426 | 3.8 |
| SDW | NLP | *Q.iari.dt.sdw.1* | AX94514240 | 1D | 11655195 | BLINK | 2.55E-08 | 0.373 | NA | 7.6 |
|  |  |  |  |  |  | MLM | 2.75E-05 | 0.373 | -0.037 | 4.6 |
|  |  | *Q.iari.dt.sdw.2* | AX94646448 | 1B | 297115087 | FARM CPU | 1.656E-06 | 0.193 | -0.034 | 5.8 |
|  |  | *Q.iari.dt.sdw.3* | AX94396598 | 7B | 46344967 | FARM CPU | 1.062E-05 | 0.127 | -0.034 | 5.0 |
|  |  | *Q.iari.dt.sdw.4* | AX94503640 | 6D | 267201017 | FARM CPU | 1.218E-05 | 0.139 | -0.032 | 4.9 |
|  |  | *Q.iari.dt.sdw.5* | AX94812403 | 5A | 18797339 | FARM CPU | 5.759E-05 | 0.114 | 0.038 | 4.2 |
|  |  | *Q.iari.dt.sdw.6* | AX94635019 | 4A | 601366905 | FARM CPU | 7.329E-05 | 0.453 | 0.026 | 4.1 |
|  |  | *Q.iari.dt.sdw.7* | AX94679648 | 4B | 601516194 | FARM CPU | 0.000167 | 0.487 | -0.021 | 3.8 |
|  | LP | *Q.iari.dt.sdw.8* | AX94638774 | 7B | 33812777 | BLINK | 7.441E-09 | 0.184 | NA | 8.1 |
|  |  |  |  |  |  | MLM | 4.794E-06 | 0.184 | -0.020 | 5.3 |
|  |  | *Q.iari.dt.sdw.9* | AX95081347 | 2D | 73426483 | BLINK | 0.0001074 | 0.089 | NA | 4.0 |
|  |  | *Q.iari.dt.sdw.10* | AX94388701 | 1A | 508929364 | BLINK | 0.0001348 | 0.184 | NA | 3.9 |
|  |  | *Q.iari.dt.sdw.11* | AX94401725 | 2D | 80820394 | BLINK | 0.000181 | 0.070 | NA | 3.7 |
|  |  | *Q.iari.dt.sdw.12* | AX94544797 | 1D | 305114395 | FARM CPU | 5.362E-07 | 0.120 | -0.029 | 6.3 |
|  |  | *Q.iari.dt.sdw.13* | AX94399951 | 1B | 352389791 | FARM CPU | 4.459E-06 | 0.104 | 0.023 | 5.4 |
|  |  |  |  |  |  | MLM | 3.66E-05 | 0.104 | 0.027 | 4.4 |
|  |  | *Q.iari.dt.sdw.14* | AX94626370 | 7B | 196908845 | FARM CPU | 3.968E-05 | 0.142 | -0.016 | 4.4 |
|  |  |  |  |  |  | MLM | 4.589E-05 | 0.142 | -0.022 | 4.3 |
|  |  | *Q.iari.dt.sdw.15* | AX94939596 | 1D | 38781551 | FARM CPU | 4.204E-05 | 0.120 | 0.019 | 4.4 |
|  |  | *Q.iari.dt.sdw.16* | AX95113278 | 5D | 527154131 | FARM CPU | 8.266E-05 | 0.104 | 0.019 | 4.1 |
|  |  | *Q.iari.dt.sdw.17* | AX94599608 | 6B | 30011466 | FARM CPU | 9.963E-05 | 0.215 | -0.009 | 4.0 |
|  |  | *Q.iari.dt.sdw.18* | AX94424121 | 5B | 517854482 | MLM | 2.208E-05 | 0.070 | 0.033 | 4.7 |
|  |  | *Q.iari.dt.sdw.19* | AX95106235 | 5D | 235652872 | MLM | 4.799E-05 | 0.139 | -0.020 | 4.3 |
|  |  | *Q.iari.dt.sdw.20* | AX94456805 | 2D | 644093426 | MLM | 7.055E-05 | 0.076 | -0.025 | 4.2 |
| RSR | NLP | *Q.iari.dt.rsr.1* | AX94475513 | 6A | 61813444 | BLINK | 6.748E-09 | 0.184 | NA | 8.2 |
|  |  |  |  |  |  | FARM CPU | 4.27E-08 | 0.184 | -0.030 | 7.4 |
|  |  |  |  |  |  | MLM | 7.712E-06 | 0.184 | -0.034 | 5.1 |
|  |  | *Q.iari.dt.rsr.2* | AX94601118 | 2D | 134792568 | BLINK | 9.935E-06 | 0.101 | NA | 5.0 |
|  |  | *Q.iari.dt.rsr.3* | AX94711378 | 7A | 5085648 | BLINK | 6.324E-05 | 0.411 | NA | 4.2 |
|  |  | *Q.iari.dt.rsr.4* | AX94449510 | 6D | 465959637 | BLINK | 0.0001696 | 0.120 | NA | 3.8 |
|  |  | *Q.iari.dt.rsr.5* | AX94648060 | 7A | 1739677 | FARM CPU | 6.046E-07 | 0.320 | -0.019 | 6.2 |
|  |  | *Q.iari.dt.rsr.6* | AX95190609 | 7A | 547573547 | FARM CPU | 6.208E-06 | 0.079 | -0.035 | 5.2 |
|  |  | *Q.iari.dt.rsr.7* | AX95021656 | 3B | 789334678 | FARM CPU | 1.045E-05 | 0.123 | 0.034 | 5.0 |
|  |  | *Q.iari.dt.rsr.8* | AX94417718 | 1B | 613233069 | FARM CPU | 1.958E-05 | 0.427 | -0.012 | 4.7 |
|  |  | *Q.iari.dt.rsr.9* | AX94864659 | 3D | 584336427 | FARM CPU | 3.04E-05 | 0.108 | 0.033 | 4.5 |
|  |  |  |  |  |  | MLM | 0.0001302 | 0.108 | 0.041 | 3.9 |
|  |  | *Q.iari.dt.rsr.10* | AX94636657 | 3D | 611255303 | FARM CPU | 3.305E-05 | 0.089 | 0.036 | 4.5 |
|  |  |  |  |  |  | MLM | 7.389E-05 | 0.089 | 0.046 | 4.1 |
|  |  | *Q.iari.dt.rsr.11* | AX94475631 | 2B | 188834804 | FARM CPU | 3.951E-05 | 0.104 | 0.025 | 4.4 |
|  |  | *Q.iari.dt.rsr.12* | AX94944176 | 4D | 69850169 | MLM | 0.0001954 | 0.092 | -0.044 | 3.7 |
|  | LP | *Q.iari.dt.rsr.13* | AX94460476 | 2D | 545937930 | BLINK | 0.0002387 | 0.291 | NA | 3.6 |
|  |  |  |  |  |  | FARM CPU | 0.0002387 | 0.291 | 0.110 | 3.6 |
|  |  | *Q.iari.dt.rsr.14* | AX94825456 | 6A | 123510075 | BLINK | 0.00028 | 0.165 | NA | 3.6 |
|  |  |  |  |  |  | FARM CPU | 0.00028 | 0.165 | -0.099 | 3.6 |
|  |  |  |  |  |  | MLM | 0.0002097 | 0.165 | -0.097 | 3.7 |
|  |  | *Q.iari.dt.rsr.15* | AX94861346 | 5D | 554349245 | BLINK | 0.0002977 | 0.180 | NA | 3.5 |
|  |  |  |  |  |  | FARM CPU | 0.0002977 | 0.180 | -0.106 | 3.5 |
|  |  | *Q.iari.dt.rsr.1* | AX94475513 | 6A | 61813444 | MLM | 8.28E-05 | 0.184 | -0.101 | 4.1 |
|  |  | *Q.iari.dt.rsr.16* | AX94488247 | 1D | 418045844 | MLM | 0.0001584 | 0.114 | -0.112 | 3.8 |
| TDW | NLP | *Q.iari.dt.sdw.1* | AX94514240 | 1D | 11655195 | BLINK | 2.035E-08 | 0.373 | NA | 7.7 |
|  |  |  |  |  |  | MLM | 3.155E-05 | 0.373 | -0.046 | 4.5 |
|  |  | *Q.iari.dt.rsr.1* | AX94475513 | 6A | 61813444 | BLINK | 0.0003284 | 0.184 | NA | 3.5 |
|  |  |  |  |  |  | FARM CPU | 0.000206 | 0.184 | -0.041 | 3.7 |
|  |  | *Q.iari.dt.sdw.2* | AX94646448 | 1B | 297115087 | FARM CPU | 2.603E-07 | 0.193 | -0.045 | 6.6 |
|  |  | *Q.iari.dt.tdw.1* | AX94577808 | 6D | 807354 | FARM CPU | 5.819E-05 | 0.187 | 0.048 | 4.2 |
|  |  | *Q.iari.dt.tdw.2* | AX94778444 | 2B | 10175521 | FARM CPU | 7.523E-05 | 0.272 | 0.035 | 4.1 |
|  |  | *Q.iari.dt.tdw.3* | AX94588315 | 1B | 125849969 | FARM CPU | 9.17E-05 | 0.446 | 0.030 | 4.0 |
|  |  | *Q.iari.dt.tdw.4* | AX94572741 | 4B | 621293 | FARM CPU | 9.489E-05 | 0.307 | 0.035 | 4.0 |
|  | LP | *Q.iari.dt.sdw.8* | AX94638774 | 7B | 33812777 | BLINK | 3.31E-10 | 0.184 | NA | 9.5 |
|  |  |  |  |  |  | MLM | 1.52E-06 | 0.184 | -0.035 | 5.8 |
|  |  | *Q.iari.dt.rsr.1* | AX94475513 | 6A | 61813444 | BLINK | 4.489E-06 | 0.184 | NA | 5.3 |
|  |  |  |  |  |  | FARM CPU | 2.638E-09 | 0.184 | -0.031 | 8.6 |
|  |  |  |  |  |  | MLM | 4.18E-05 | 0.184 | -0.032 | 4.4 |
|  |  | *Q.iari.dt.tdw.5* | AX94733613 | 2B | 768613714 | BLINK | 0.0002227 | 0.269 | NA | 3.7 |
|  |  | *Q.iari.dt.tdw.6* | AX94621027 | 7A | 558082185 | FARM CPU | 8.039E-07 | 0.203 | 0.028 | 6.1 |
|  |  | *Q.iari.dt.tdw.7* | AX94734828 | 7D | 210393100 | FARM CPU | 5.014E-06 | 0.066 | -0.040 | 5.3 |
|  |  |  |  |  |  | MLM | 0.0001404 | 0.066 | -0.038 | 3.9 |
|  |  | *Q.iari.dt.tdw.8* | AX94530943 | 7B | 587910543 | FARM CPU | 1.871E-05 | 0.212 | -0.016 | 4.7 |
|  |  | *Q.iari.dt.tdw.9* | AX94426211 | 5B | 457128036 | FARM CPU | 1.923E-05 | 0.443 | -0.014 | 4.7 |
|  |  | *Q.iari.dt.sdw.14* | AX94626370 | 7B | 196908845 | FARM CPU | 2.6E-05 | 0.142 | -0.026 | 4.6 |
|  |  |  |  |  |  | MLM | 2.93E-05 | 0.142 | -0.036 | 4.5 |
|  |  | *Q.iari.dt.tdw.10* | AX94884567 | 2A | 760619493 | FARM CPU | 4.591E-05 | 0.351 | 0.014 | 4.3 |
|  |  | *Q.iari.dt.tdw.11* | AX95014147 | 5D | 509516182 | FARM CPU | 9.11E-05 | 0.060 | 0.056 | 4.0 |
|  |  | *Q.iari.dt.sdw.20* | AX94456805 | 2D | 644093426 | FARM CPU | 0.0001271 | 0.076 | -0.038 | 3.9 |
| TPC | NLP | *Q.iari.dt.tpc.1* | AX94905933 | 7A | 67671413 | BLINK | 0.0001613 | 0.282 | NA | 3.8 |
|  |  |  |  |  |  | FARMCPU | 0.0001613 | 0.282 | -0.553 | 3.8 |
|  |  | *Q.iari.dt.tpc.2* | AX94584110 | 3D | 370534927 | BLINK | 0.0002361 | 0.206 | NA | 3.6 |
|  |  |  |  |  |  | FARMCPU | 0.0002361 | 0.206 | -0.744 | 3.6 |
|  | LP | *Q.iari.dt.tpc.3* | AX94397869 | 3A | 543712481 | BLINK | 0.0001539 | 0.278 | NA | 3.8 |
|  |  |  |  |  |  | FARM CPU | 0.0001539 | 0.278 | 0.203 | 3.8 |
|  |  | *Q.iari.dt.tpc.4* | AX94978370 | 3A | 532846802 | BLINK | 0.000165 | 0.301 | NA | 3.8 |
|  |  |  |  |  |  | FARM CPU | 0.000165 | 0.301 | 0.210 | 3.8 |
|  |  | *Q.iari.dt.tpc.5* | AX94935938 | 3D | 397166861 | BLINK | 0.0001671 | 0.342 | NA | 3.8 |
|  |  |  |  |  |  | FARM CPU | 0.0001671 | 0.342 | 0.218 | 3.8 |
| TPU | NLP | *Q.iari.dt.tpu.1* | AX94815880 | 1D | 10432903 | BLINK | 0.0002026 | 0.203 | NA | 3.7 |
|  |  |  |  |  |  | FARM CPU | 0.0002026 | 0.203 | -0.427 | 3.7 |
|  |  | *Q.iari.dt.sdw.3* | AX94396598 | 7B | 46344967 | BLINK | 0.0002379 | 0.127 | NA | 3.6 |
|  |  |  |  |  |  | FARM CPU | 0.0002379 | 0.127 | -0.498 | 3.6 |
|  |  |  |  |  |  | MLM | 0.0002778 | 0.127 | -0.487 | 3.6 |
|  |  | *Q.iari.dt.sdw.1* | AX94514240 | 1D | 11655195 | BLINK | 0.0002397 | 0.373 | NA | 3.6 |
|  |  |  |  |  |  | FARM CPU | 0.0002397 | 0.373 | -0.353 | 3.6 |
|  |  |  |  |  |  | MLM | 0.0003389 | 0.373 | -0.312 | 3.5 |
|  | LP | *Q.iari.dt.tpc.3* | AX94397869 | 3A | 543712481 | BLINK | 7.442E-05 | 0.278 | NA | 4.1 |
|  |  |  |  |  |  | FARM CPU | 7.442E-05 | 0.278 | 0.047 | 4.1 |
|  |  | *Q.iari.dt.tpu.2* | AX94861851 | 3A | 544385295 | BLINK | 9.869E-05 | 0.364 | NA | 4.0 |
|  |  |  |  |  |  | FARM CPU | 9.869E-05 | 0.364 | 0.045 | 4.0 |
|  |  | *Q.iari.dt.tpu.3* | AX94713349 | 3A | 517079885 | BLINK | 0.0001165 | 0.291 | NA | 3.9 |
|  |  |  |  |  |  | FARM CPU | 0.0001165 | 0.291 | 0.044 | 3.9 |
|  |  | *Q.iari.dt.tpu.4* | AX94611921 | 3D | 389912635 | BLINK | 0.0001408 | 0.266 | NA | 3.9 |
|  |  |  |  |  |  | FARM CPU | 0.0001408 | 0.266 | 0.045 | 3.9 |
| PUtE | NLP | *Q.iari.dt.tpc.2* | AX94584110 | 3D | 370534927 | BLINK | 7.515E-05 | 0.206 | NA | 4.1 |
|  |  |  |  |  |  | FARM CPU | 7.515E-05 | 0.206 | 0.025 | 4.1 |
|  |  | *Q.iari.dt.pute.1* | AX94702674 | 3D | 370534850 | BLINK | 0.0002226 | 0.104 | NA | 3.7 |
|  |  |  |  |  |  | FARM CPU | 0.0002226 | 0.104 | -0.039 | 3.7 |
|  | LP | *Q.iari.dt.pute.2* | AX94655055 | 5B | 550211719 | BLINK | 2.821E-06 | 0.114 | NA | 5.5 |
|  |  |  |  |  |  | FARM CPU | 2.821E-06 | 0.114 | 0.276 | 5.5 |
|  |  |  |  |  |  | MLM | 5.826E-05 | 0.114 | 0.259 | 4.2 |
|  |  | *Q.iari.dt.pute.3* | AX94590370 | 3D | 490839541 | BLINK | 0.0001163 | 0.320 | NA | 3.9 |
|  |  |  |  |  |  | FARM CPU | 0.0001163 | 0.320 | -0.134 | 3.9 |
|  |  |  |  |  |  | MLM | 0.0001767 | 0.320 | -0.125 | 3.8 |

**Supplementary Table 4: List of Marker-Trait Associations (MTAs) detected in three models: BLINK, FARM CPU, and MLM**

| QTLs | SNP | Chromosome | Position | Model |
| --- | --- | --- | --- | --- |
| *Q.iari.dt.tla.1* | AX94470386 | 7B | 6.48E+08 | BLINK |
|  |  |  |  | FARM CPU |
|  |  |  |  | MLM |
| *Q.iari.dt.tla.3* | AX94770913 | 6A | 6.12E+08 | BLINK |
|  |  |  |  | FARM CPU |
|  |  |  |  | MLM |
| *Q.iari.dt.tla.4* | AX95190390 | 1B | 48689512 | BLINK |
|  |  |  |  | FARM CPU |
|  |  |  |  | MLM |
| *Q.iari.dt.chl.4* | AX94597699 | 6B | 36206328 | BLINK |
|  |  |  |  | FARM CPU |
|  |  |  |  | MLM |
| *Q.iari.dt.chl.5* | AX95241386 | 6A | 18706523 | BLINK |
|  |  |  |  | FARM CPU |
|  |  |  |  | MLM |
| *Q.iari.dt.chl.6* | AX94702861 | 6D | 21051029 | BLINK |
|  |  |  |  | FARM CPU |
|  |  |  |  | MLM |
| *Q.iari.dt.chl.8* | AX95230097 | 6D | 21981934 | BLINK |
|  |  |  |  | FARM CPU |
|  |  |  |  | MLM |
| *Q.iari.dt.rsr.1* | AX94475513 | 6A | 61813444 | BLINK |
|  |  |  |  | FARM CPU |
|  |  |  |  | MLM |
| *Q.iari.dt.rsr.14* | AX94825456 | 6A | 1.24E+08 | BLINK |
|  |  |  |  | FARM CPU |
|  |  |  |  | MLM |
| *Q.iari.dt.rsr.1* | AX94475513 | 6A | 61813444 | BLINK |
|  |  |  |  | FARM CPU |
|  |  |  |  | MLM |
| *Q.iari.dt.sdw.3* | AX94396598 | 7B | 46344967 | BLINK |
|  |  |  |  | FARM CPU |
|  |  |  |  | MLM |
| *Q.iari.dt.sdw.1* | AX94514240 | 1D | 11655195 | BLINK |
|  |  |  |  | FARM CPU |
|  |  |  |  | MLM |
| *Q.iari.dt.pute.2* | AX94655055 | 5B | 5.5E+08 | BLINK |
|  |  |  |  | FARM CPU |
|  |  |  |  | MLM |
| *Q.iari.dt.pute.3* | AX94590370 | 3D | 4.91E+08 | BLINK |
|  |  |  |  | FARM CPU |
|  |  |  |  | MLM |

**Supplementary Table 5:** Analysis of Variance for the traits under non-limiting (5A) and (5B) limiting phosphorus

5A

| **Source of Variation** | **MSS** | | **P value** |
| --- | --- | --- | --- |
|  | **Genotype** | **Error** |  |
| **DF** | 157 | 316 |  |
| TLA | 172.716 | 14.6861 | *** |
| Chl | 52.2952 | 1.5136 | *** |
| SDW | 0.0029 | 0 | *** |
| RSR | 0.0781 | 0.0008 | *** |
| TDW | 0.0066 | 0.0001 | *** |
| TPC | 0.685 | 0.0035 | *** |
| TPU | 0.0375 | 0.0003 | *** |
| PUtE | 0.3204 | 0.003 | *** |

5B

| **Source of Variation** | **MSS** | | **P value** |
| --- | --- | --- | --- |
|  | **Genotype** | **Error** |  |
| **DF** | 157 | 316 |  |
| TLA | 756.49 | 38.8937 | *** |
| Chl | 33.9234 | 1.3847 | *** |
| SDW | 0.0177 | 0 | *** |
| RSR | 0.0067 | 0.0001 | *** |
| TDW | 0.0285 | 0.0001 | *** |
| TPC | 4.8062 | 0.0169 | *** |
| TPU | 1.8052 | 0.0035 | *** |
| PUtE | 0.0046 | 0 | *** |
